# Supplementary material for: Selection by a panel of clinicians and family representatives of important early morbidities associated with paediatric cardiac surgery suitable for routine monitoring using the nominal group technique and a robust voting process
Source: BMJ Open. 2017 May 29;7(5):e014743. doi: 10.1136/bmjopen-2016-014743 (PMC5729972; doi:10.1136/bmjopen-2016-014743)
Supplement: Supplementary appendices [file bmjopen-2016-014743supp001.pdf]

Appendix 1

Voting process used in selection

The voting process used as part of the nominal group technique deployed was based on the algorithm presented in *Utley M, Gallivan S, Mills M, Mason M, Hargraves C. A consensus process for identifying a prioritised list of study questions. Health Care Manag Sci. 2007 Feb;10(1):105–10*. A short summary is presented here.

In a secret ballot, each panellist ranked the candidate options in order of descending importance. They were permitted to use tied ranks and were not obliged to rank every option. For each and every possible pair of options (candidate morbidities A and B say) we determined whether at least as many participants preferred option A to option B as preferred option B to option A. We then used the analysis summarised in Box 1 to identify group preferences among the set of candidate morbidities.

In the first panel meeting, the rankings supplied by individual panellists were entered into an Excel spreadsheet and a VBA Macro written by MU and CP was used to conduct the analysis. For the online poll conducted after the second panel meeting, we used the web-based tool available at www.crankit.io.

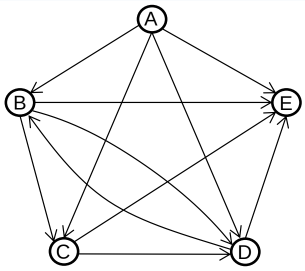

Step 1

Draw an arrow from option A to B if at least as many participants ranked A higher than B as ranked B higher than A. Repeat process in each direction for all pairs of options.

In this example there is an arrow in both directions between B and D, indicating that exactly as many participants preferred B to D as preferred D to B…

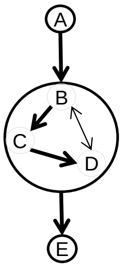

Step 2

Identify the hierarchy of subsets of options. Within a subset there is no clear group ranking of options.

…there is a majority preference for A above every other option and E is least favoured. The cycle of arrows between B, C and D indicates that there is no unambiguous ranking of these 3...

**Rank before tie-breaks**	**Option**	**Rank after tie-breaks**
1	A	1
2	B	2
2	C	3
2	D	4
5	E	5

Step 3

Try to rank options within subsets by first counting the arrows leaving each option within that subset and then, if there are ties, counting just those arrows between tied options.

… there are two arrows leaving B and just 1 leaving each of C and D, so the first tie-break places B above C and D. Then, considering just C and D, the second tie break places C above D.

Box 1; explanation of the robust group ranking process used in the nominal group approach employed by the selection panel.

Appendix 2

Panel composition

Panellist	Role on panel
1*	Family representative
2	Heart surgeon
3†	Intensive care doctor
4†	Cardiologist
5	Intensive care doctor
6†	Paediatrician
7	Family representative
8*	Paediatrician
9	Intensive care nurse
10*	Heart surgeon
11	Family representative
12†	Clinical nurse specialist
13	Heart surgeon
14†	Cardiologist
15†	Clinical psychologist

Table A2 Members of the selection panel. * indicates members who could not attend the 1st panel meeting, † those who could not attend the 2nd meeting. Further details are available at http://www.gosh.nhs.uk/medical-information/clinical-specialties/cardiothoracic-surgery-information-parents-and-visitors/why-we-do-research/complications-after-heart-surgery-children.

Appendix 3: The initial 66 terms under consideration in selection panel 1.

anxiety/fear/aggression	prolonged ventilation
problems feeding	complications during surgery
reflux/vomiting	developmental delay
pleural effusion	financial difficulties
swallowing & choking	lack of support at home
global permanent neurological impairment	laryngopharyngeal dysfunction
Mediastinitis	vocal cord dysfunction
myocardial ischemia	delay in establishing feeding
focal permanent neurological impairment	brain damage
sensory neural deafness	low cardiac output
brain injury	Sepsis
cardiac arrest	gastrosomy
renal failure/insufficiency	massive haemorrhage
length of ICU stay	thrombosis
post-traumatic stress disorder	impaired cognitive function more than a month after surgery
ECMO / mechanical support	quality of life
communication	prolonged hospital length of stay
hospital procedural anxiety	permanent pacemaker
adjustment to difference	long term renal support
Attachment	any serious incident during patient stay
neurological insult	cost per quality measure
Chylothorax	seizures
acute kidney injury	stroke
necrotising enterocolitis	sternal wound infection
junctional ectopic tachycardia (JET)	psychological/behavioural issues
pulmonary hypertension	growth retardation
SVC obstruction	tracheostomy
swelling of head & neck	diaphragmatic palsy
chest exploration	delayed chest closure
thrombosis of venous pathways	respiratory infection
irreversible neurological damage	wrong clinical decision
renal replacement therapy	wrong clinical diagnosis
unplanned reoperation within 30 days	death before surgery/delayed surgery

Appendix 4 - The summary of the feasibility of defining and measuring in routine practice (as judged by the definition panel) vs importance shown to the selection panel at their second meeting.

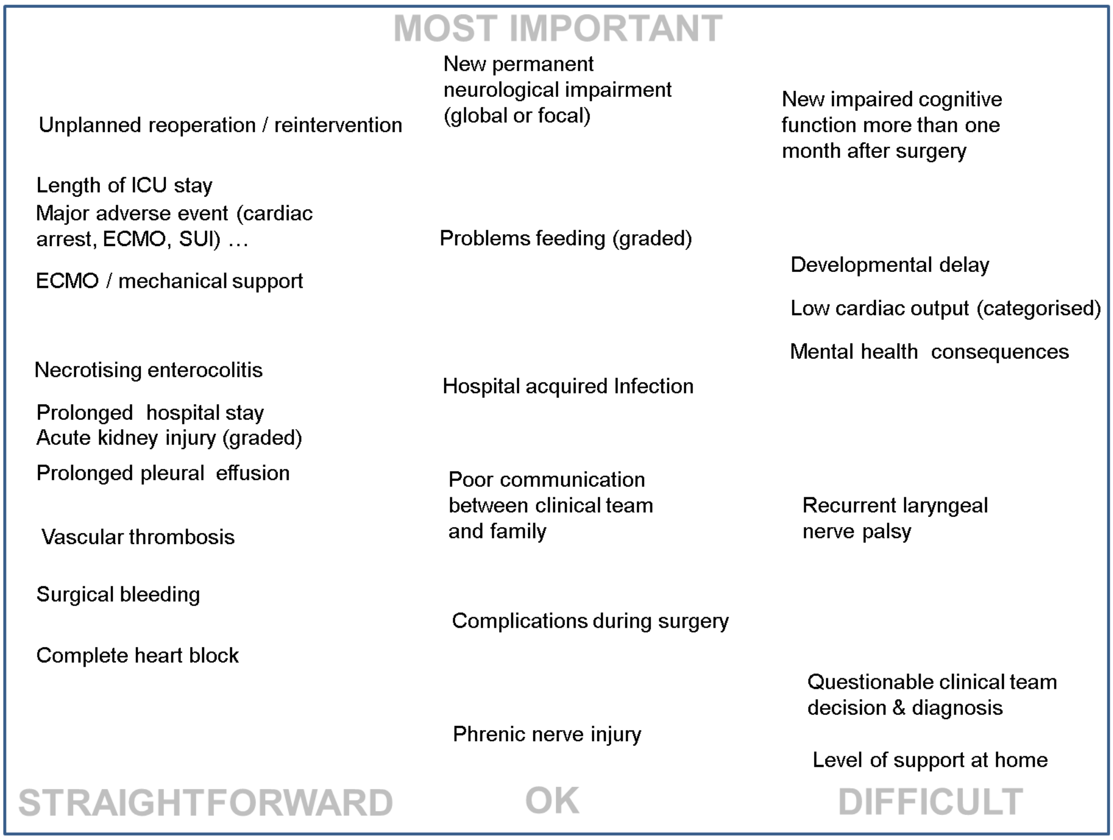

Appendix 5

Graphics shown to the selection panel at the second meeting on the relationships between candidate morbidities (figures A1-A3).

Figure A1

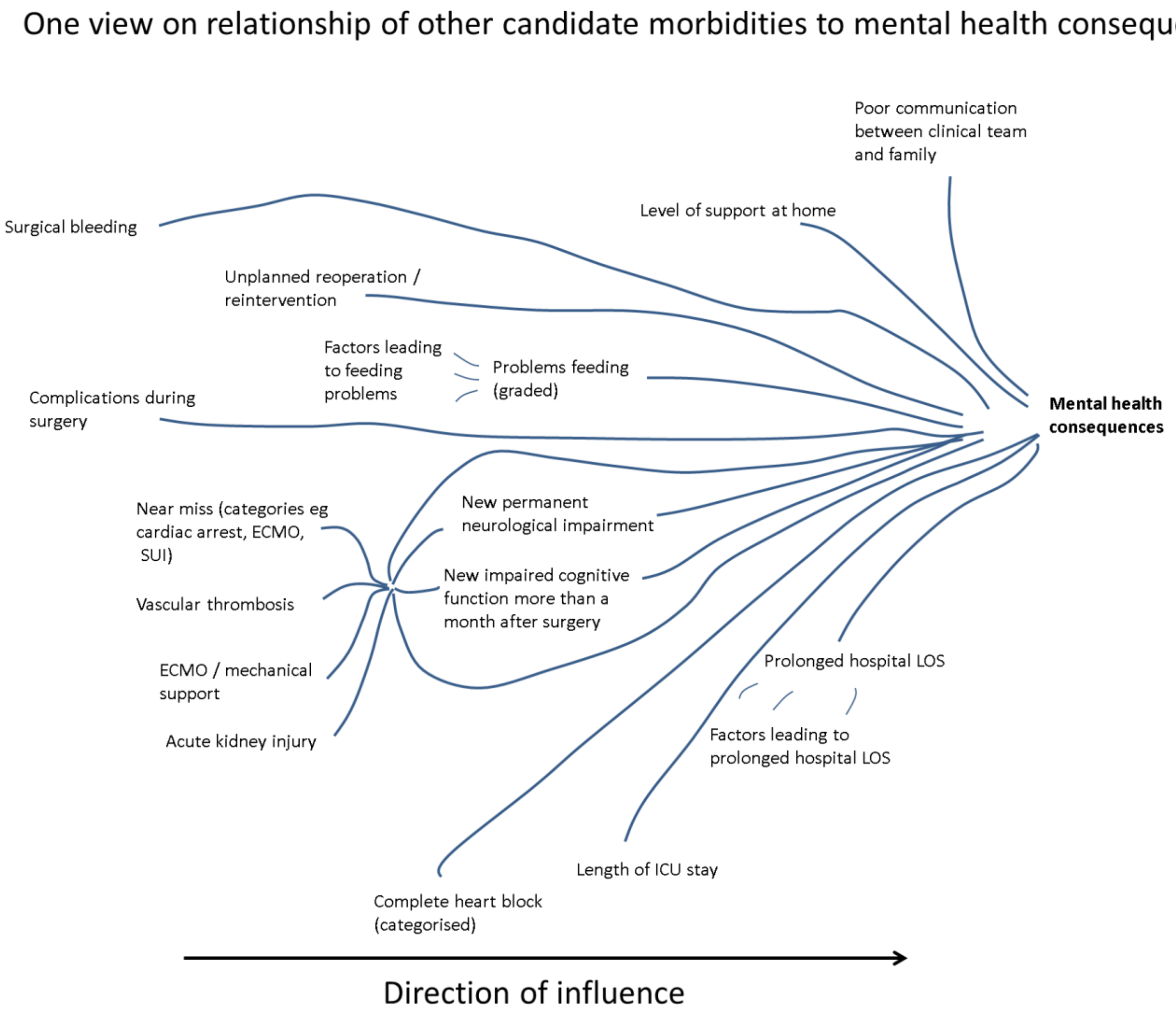

Figure A2

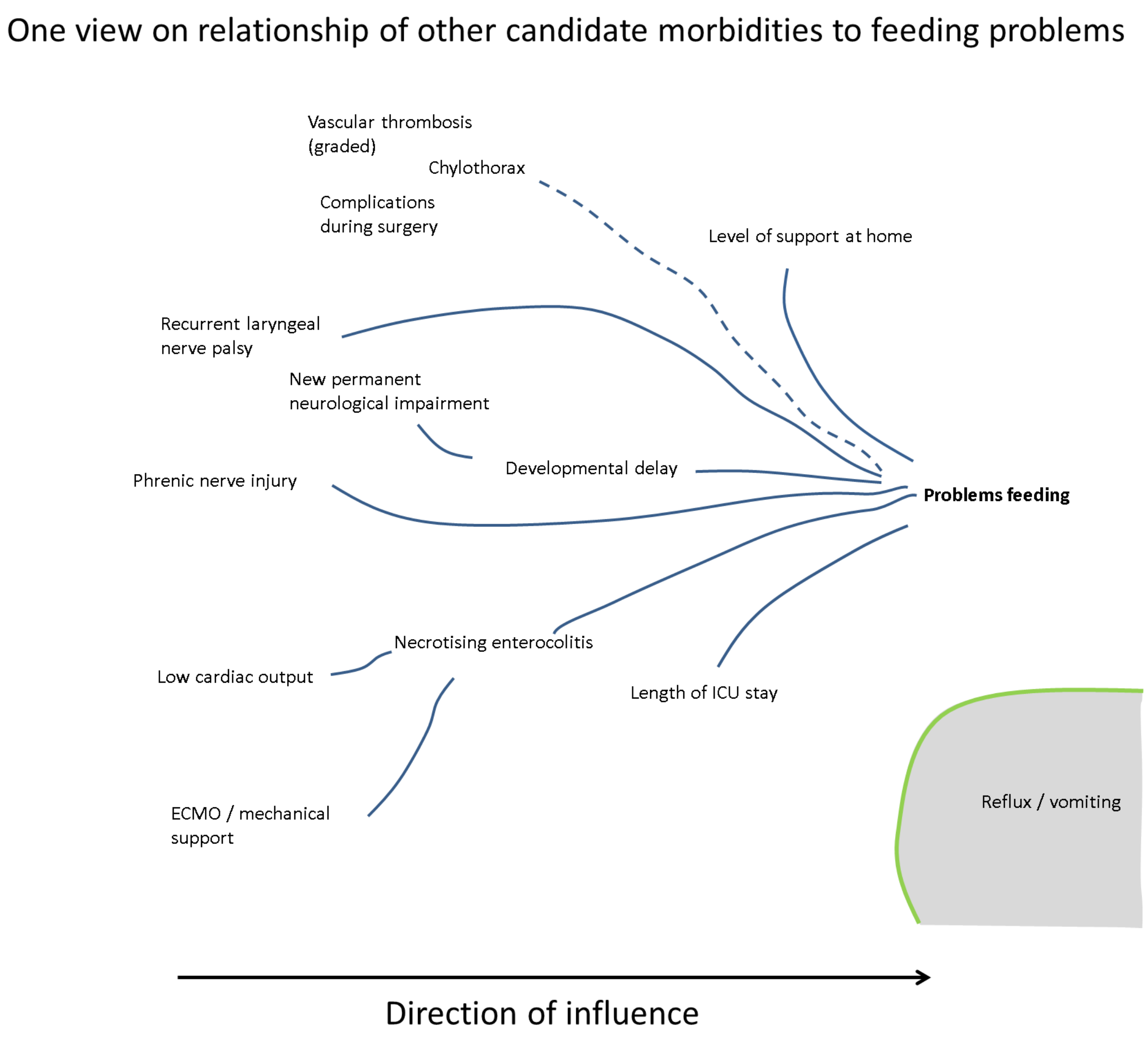
One view on relationship of other candidate morbidities to feeding problems
Vascular thrombosis (graded)
Chylothorax
Complications during surgery
Level of support at home
Recurrent laryngeal nerve palsy
New permanent neurological impairment
Developmental delay
Phrenic nerve injury
Problems feeding
Necrotising enterocolitis
Low cardiac output
Length of ICU stay
ECMO / mechanical support
Reflux / vomiting
Direction of influence

Figure A3

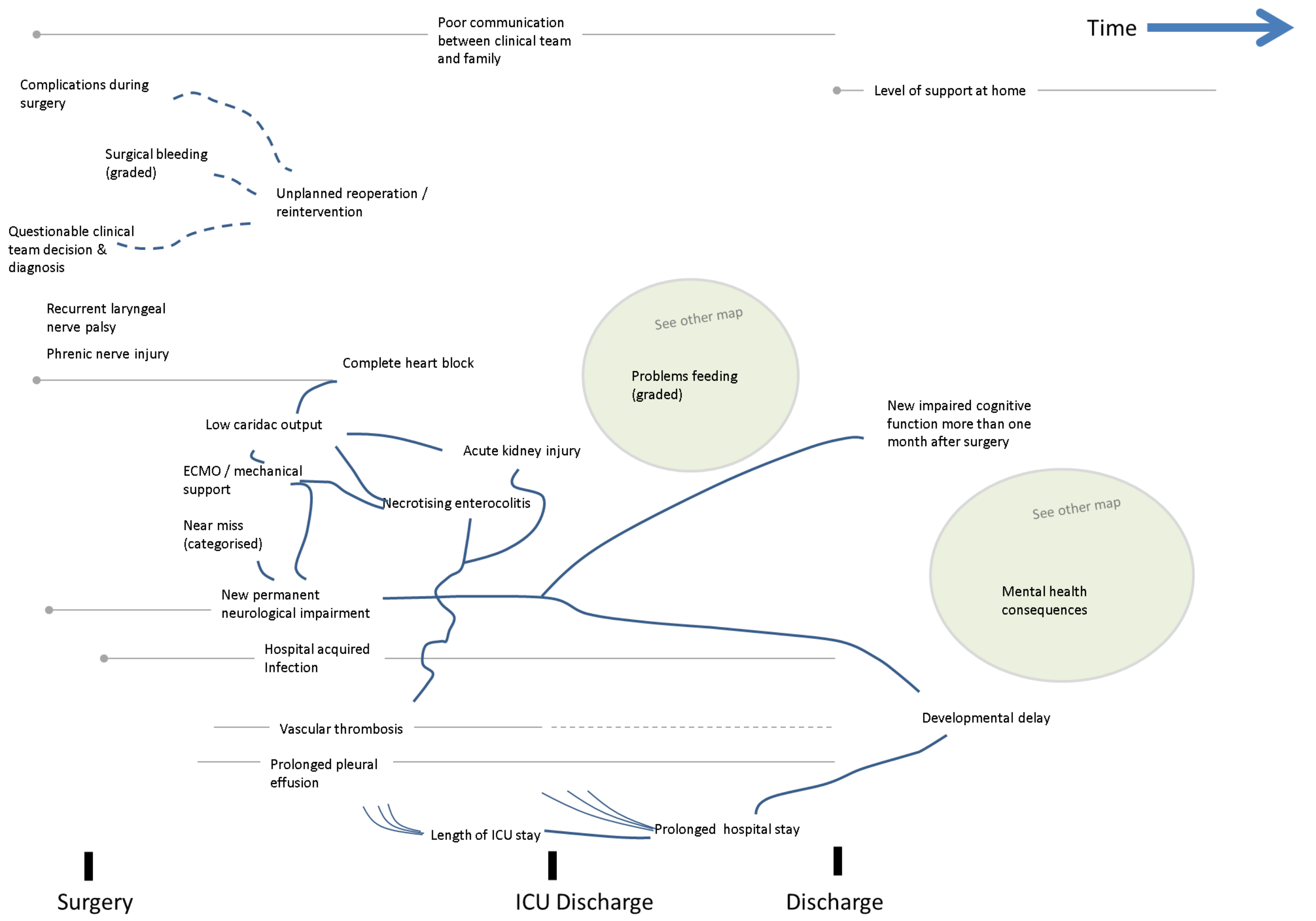

One view of whole picture (relationships of candidate morbidities to each other)
