# Supplementary material for: Selection by a panel of clinicians and family representatives of important early morbidities associated with paediatric cardiac surgery suitable for routine monitoring using the nominal group technique and a robust voting process
Source: BMJ Open. 2017 May 29;7(5):e014743. doi: 10.1136/bmjopen-2016-014743 (PMC5729972; doi:10.1136/bmjopen-2016-014743)
Supplement: Author's manuscript [file bmjopen-2016-014743.draft_revisions.pdf]

Selection by a panel of clinicians and family representatives of important early morbidities associated with paediatric cardiac surgery suitable for routine monitoring.

Journal:	*BMJ Open*
Manuscript ID	bmjopen-2016-014743
Article Type:	Research
Date Submitted by the Author:	16-Oct-2016
Complete List of Authors:	Pagel, Christina; UCL, Clinical Operational Research Unit<br>Brown, Katherine; Great Ormond Street Hospital for Children, Cardiorespiratory Division<br>Macleod, Isobel; Royal Hospital for Children<br>Jepps, Helen; Bradford Teaching Hospitals NHS Foundation Trust<br>Wray, Jo; Great Ormond Street Hospital for Children, Cardiorespiratory Division<br>Chigaru, Linda; Great Ormond Street Hospital for Children, Cardiorespiratory Division<br>Mclean, Andrew; Royal Hospital for Children<br>Treasure, Tom; UCL, CORU Mathematics<br>Tsang, Victor; Great Ormond Street Hospital for Children, Cardiorespiratory Division<br>Utley, Martin; University College London, Clinical Operational Research Unit, Department of Mathematics
**Primary Subject Heading**:	Surgery
Secondary Subject Heading:	Patient-centred medicine, Research methods, Health services research
Keywords:	Paediatric cardiac surgery < PAEDIATRIC SURGERY, AUDIT, Quality in health care < HEALTH SERVICES ADMINISTRATION & MANAGEMENT

SCHOLARONE™
Manuscripts

Selection by a panel of clinicians and family representatives of important early morbidities associated with paediatric cardiac surgery suitable for routine monitoring.

Authors: Christina Pagel, Katherine L Brown, Isobel McLeod, Helen Jepps, Jo Wray, Linda Chigaru, Andrew McLean, Tom Treasure, Victor Tsang, Martin Utley

Contributors: Rehana Ahmed, David Barron, Jane Cassidy, Kate English, Allan Goldman, Samantha Johnson, Ravi Kumar, Andrew Parry, Samana Schwank, Emma Simmonds, Rob Tulloh, Emma Twigg.

Email address for corresponding author: m.utley@ucl.ac.uk

Abstract

Objective: With survival following paediatric cardiac surgery improving, the attention of quality assurance and improvement initiatives is shifting to long-term outcomes and early surgical morbidities. We wanted to involve family representatives and a range of clinicians in selecting the morbidities to be measured in a major UK study.

Setting: Paediatric cardiac surgery services in the UK.

Participants: We convened a panel comprising family representatives, paediatricians from referring centres and surgeons and other clinicians from surgical centres.

Primary and secondary outcome measures: Using the Nominal Group Technique augmented by a robust voting process to identify group preferences, suggestions for candidate morbidities were elicited, discussed, ranked and then shortlisted. The shortlist was passed to a clinical group that provided a view on the feasibility of monitoring each shortlisted morbidity in routine practice. The panel then met again to select a prioritised list of morbidities for further study, with the list finalised by the clinical group and chief investigators.

Results: At the first panel meeting, 66 initial suggestions were made, with this reduced to a shortlist of 24 after two rounds of discussion, consolidation and voting. At the second meeting, this shortlist was reduced to 10 candidate morbidities. Two were dropped on grounds of feasibility and replaced by another the panel considered important. The final list of 9 morbidities included indicators of organ damage, acute events and feeding problems. Family representatives and clinicians from outside tertiary centres brought some issues to greater prominence than if the panel had consisted solely of tertiary clinicians or study investigators.

Conclusion: The inclusion of patient and family perspectives in identifying metrics for use in monitoring a specialised clinical service is challenging but feasible and can broaden notions of quality and how to measure it.

Word counts: Abstract: 287 Main text**:** 3884.

For peer review only - http://bmjopen.bmj.com/site/about/guidelines.xhtml

Strengths and limitations

There is an increasing focus from clinicians, families and commissioners on early morbidity following paediatric cardiac surgery.

There is a growing recognition of the importance of involving patients and families in the choice of outcome measures to use in research, quality improvement and quality assurance.

We used the Nominal Group Technique, augmented by a robust voting process to incorporate the perspectives of family representatives and different professional groups in selecting early surgical morbidities they felt important to monitor in routine practice.

There was some tension between selecting morbidities clearly attributable to the surgical act and morbidities that are important to families but can be considered to "come with the territory" of congenital heart disease and its management.

The incidence and impact of the 9 selected morbidities are now being measured as part of a national study.

Introduction

Early mortality following paediatric cardiac surgery, defined as death within 30 days of surgery or death prior to discharge home, has been the focus of many research studies (1–10), audit initiatives (6,11–14) and, particularly in the UK, of public scrutiny (1,15–20) over recent decades. However, with early mortality having fallen to 2-3% (21), attention has shifted to longer term outcomes and to broadening the assessment of early outcomes to include early morbidities.

Reporting of early morbidities associated with paediatric cardiac surgery has been driven largely by the data available within the databases of professional societies (12) or those available within datasets curated by individual clinical teams (22). Quality assurance initiatives rooted in such datasets benefit greatly from considerable effort, often over many years, to agree definitions of the outcomes collected and design data collection processes. However, it is inevitable that the datasets agreed upon, constructed and curated by clinicians (as individuals or via professional societies) focus largely on outcomes considered important from the perspective of that clinician or professional group. Research in other specialties has shown that patients and carers can have quite different perceptions to clinicians on what outcomes are important to monitor as part of service evaluation (23).

We report here on a process used to select early morbidities as part of a study to identify and then measure the incidence and impact of important early morbidities among paediatric cardiac surgery patients (NIHR HS&DR 12/5005/06 (24)). A key aim of our work was to incorporate a broad set of perspectives, including those from family representatives and professionals from different sectors on what early morbidities were important to monitor in routine practice.

For peer review only - http://bmjopen.bmj.com/site/about/guidelines.xhtml

Methods

Overview

Figure 1 gives an overview of the role of our selection process within the wider study. A panel of clinicians and patient representatives met twice to shortlist and then to select early morbidities, the incidence and impact of which they considered important to measure. As shown in Figure 1, our selection process was linked to a parallel process of defining potential morbidities. The shortlist of morbidities produced after the first meeting of the selection panel was considered by a separate group composed entirely of clinicians. This definition panel provided a view on the feasibility of defining, measuring and routinely monitoring each shortlisted morbidity, which informed the second selection meeting.

Figure 1 HERE

Composition of the selection panel

In forming the panel, we aimed to include clinicians from surgical centres, referring hospitals and primary care as well as family representatives. We wanted a panel of enough people to provide a range of perspectives while being manageable.

The panel comprised 15 people: 3 family representatives, 3 paediatric cardiac surgeons, 2 paediatric intensive care doctors, 2 paediatric cardiologists, 2 paediatricians, a paediatric intensive care nurse, a clinical nurse specialist and a clinical psychologist with experience of working with children with congenital heart disease and their families. The panel was chaired by a cardiothoracic surgeon with extensive experience of chairing multi-disciplinary panels. We tried, but did not manage, to recruit a GP to the panel.

With the permission of panel members, both selection panel meetings were recorded and professionally transcribed. Each selection panel meeting also had a pre-determined seating plan to ensure that people from similar specialities were not grouped together.

Selection panel meeting 1: shortlisting

The aim of the first meeting was to identify a shortlist of 15-20 candidate morbidities that would then be considered by the definitions group. Prior to the meeting, the panel was supplied with an extensive list of candidate morbidities identified through:

- an ongoing systematic review conducted as part of our wider programme of research;
- three facilitated focus groups held in different UK cities with parents recruited by the Children's Heart Federation;
- an online forum for patients and families hosted on the website of the Children's Heart Federation.

For peer review only - http://bmjopen.bmj.com/site/about/guidelines.xhtml

The focus groups and literature review will be the subject of other publications. The panel was also sent an abridged version of the study protocol, a description of the role of the selection panel and an agenda. Each panellist was asked to identify among or beyond this list of candidate morbidities, those they judged most important to monitor routinely according to a deliberately broad working definition of a surgical morbidity as:

> *Any health or emotional problem that arose as a result of the fact of surgery (whether directly caused by surgery/post-operative care or not).*

For the first meeting, panellists were requested not to censor their suggestions on grounds of the perceived difficulty of definition or measurement and it was stressed that another group would be making these judgements.

We used the nominal group technique (25,26) augmented by a robust voting process to determine group rankings of morbidities. The nominal group technique is designed to reduce the influence of perceived power differentials and of dominant personalities on group decision making whilst retaining the benefit of discussion absent from other systematic approaches to group decision making such as Delphi (27,28). We inferred group preferences from individual rankings using a method developed by Utley et al (29) that is briefly described in appendix 1.

Structure of the first panel meeting

Each panellist was given the opportunity to speak uninterrupted for two minutes on the morbidities they considered important. Each suggestion was entered onto a spreadsheet which was projected in the meeting room to ensure accurate transcription. The panel was then given the opportunity to add to this initial list if they thought something important had been missed.

The Chair led a process of identifying suggestions that fell outside the working definition above, duplication among suggestions and merging of closely related suggestions. There was then a secret ballot in which panellists were asked individually to rank the resulting list of candidate suggestions in order of descending importance. The voting process and the method for generating group preferences from individual ranking data are described further in appendix 1.

During a scheduled break, the group preferences were calculated from the individual rankings by two of the authors (CP, MU) who did not have a vote on which morbidities to measure. After the break, the group preferences were fed back to the panel. The Chair then led a second round of discussion, focussing on the group preferences and giving panellists the opportunity to argue for specific morbidities being given greater importance, and for the group to further consolidate the list of morbidities.

There was a second round of secret ranking followed by feedback of group preferences prior to a consensus being sought as to the prioritised shortlist of 15 to 20 morbidities to be passed to the separate definitions panel for an assessment on the feasibility of defining, measuring and monitoring each in routine practice.

For peer review only - http://bmjopen.bmj.com/site/about/guidelines.xhtml

Causal mapping

Following the first selection group meeting, it was decided that it might be useful for non-clinical members of the panel to have an accessible summary of any causal relationships among candidate morbidities so that, when choosing the best set of morbidities to monitor, any overlap or redundancy among candidates could be accounted for. To this end, a set of causal mapping exercises was conducted by one of the facilitative team (MU) separately with two of the panellists (IM and HJ). In each exercise, cards representing shortlisted morbidities were placed on a large sheet of paper and arranged left to right with lines drawn to indicate potential causal relationships. Photographs were taken of these causal maps which were then converted to diagrams.

For peer review only

Selection panel meeting 2: incorporating feasibility and overlap.

Prior to the second meeting, panellists were provided with a pack of materials containing:

- a summary of any estimates of incidence and impact of candidate morbidities from the systematic review
- the judgment of the definition panel on the feasibility of defining, measuring and monitoring routinely each candidate morbidity
- summary of potential long term impacts of each candidate morbidity from the definition panel
- a summary of the parent and family focus groups and the online forum
- the diagrams generated through the causal mapping exercise.
- minutes from the first meeting
- a statement of the purpose of the second meeting and its agenda

At the beginning of the second meeting the panel were given a brief reminder of the scope of the overall project, the remit of the selection panel and the timescales we were working to.

The panel were tasked with narrowing the list of shortlisted candidates to a selection of 6-10, the incidence and impact of which would be measured in five centres over 18 months (30). It was explained that the upper limit of 10 morbidities was due to the sample size required for measuring the impact of distinct morbidities. It was explained that we could measure just the incidence of other morbidities if possible from routine data.

Panellists were also alerted to the (then) recently launched NHS England consultation on the future of Children's Heart Services in England, which highlighted the possibility of future national audit of surgical morbidities.

Panellists were asked to consider the following:

- *Feasibility of measurement, including timescales*. Given our plans to measure the impact of morbidities, the project team stressed that selected morbidities needed to be identifiable in a timely manner.

- *Overlap and redundancy among selected morbidities*. The project team made the point that selecting morbidities that almost always occur with other selected morbidities would pose problems in terms of measuring their individual impact. It highlighted that length of stay measures are particularly problematic in this respect.

- *Incidence*. Selected morbidities needed to have an incidence of at least 1.5-2% for us to measure their impact over 18 months due to sample-size considerations.

A summary of the judgements of the definition panel was presented to the panel, consisting of an array showing the shortlisted candidates placed vertically based on the group ranking of importance from meeting 1 and horizontally in terms of the feasibility of monitoring that morbidity.

The panel were then asked in a secret ballot to nominate morbidities for exclusion without further discussion and others for inclusion without further discussion. The panel discussed the remaining morbidities as a group.

After the panel meeting, a written summary of the discussion was circulated and an online poll conducted to obtain the group ranking of importance among the shortlisted candidate morbidities. The poll was conducted to elicit the views of panellists who weren't able to attend and to identify replacement morbidities if any of those selected were judged to be infeasible to monitor in routine practice. We used the online voting tool at www.crankit.io, which uses the same algorithm for robustly inferring group preferences from individual preference data that was used in the selection meetings and that is outlined in appendix 1.

Final review of selected morbidities by definition panel and chief investigators

The selected morbidities were then reviewed by the definition panel, which could veto inclusion of a selected morbidity if, after careful consideration and discussion with the chief investigators (VT, KB), it was deemed infeasible to define and monitor in routine practice.

RESULTS

The panel convened is given at appendix 2. Of the 15 panellists, 3 could not attend the first meeting and 6 could not attend the second. An overview of the results of the selection process is given in figure 2.

FIGURE 2 HERE

For peer review only - http://bmjopen.bmj.com/site/about/guidelines.xhtml

First panel meeting:

At the first meeting, 66 morbidity terms were suggested during the round of 2 minute contributions from each panellist (see appendix 3). In the discussion that followed, 7 terms were removed as being irrelevant to our study, related to impacts of post-surgical morbidity that would either be measured as part of the our empirical study or were too long-term in nature to be captured within our study, or redundant given other terms suggested.

Of the remaining 59, 7 were accepted as candidate morbidities as they were and a further 5 were simply relabelled. The remaining 47 terms were mapped onto 16 groups of 2 – 8 terms, with each group considered to relate to a sufficiently similar phenomenon for them to be a single candidate morbidity. Members of the panel confirmed that it wanted the term "ECMO / Mechanical Support" to feature on its own as well as being an indicator of a "Major Adverse Event". A new term ("liver injury") was added as a candidate at this stage with the agreement of the panel. This gave 29 candidate morbidities.

These candidate morbidities and the group ranking of importance among them in the first round of voting are shown in table 1. It is worth noting that, in this first round of voting, the group ranked "new global permanent neurological impairment" as the most important morbidity. After that there was a group of 22 candidate morbidities that could only be separated by applying tie-breaks, with the other six candidate morbidities (including "necrotising enterocolitis") ranked as less important.

TABLE 1 HERE

In the discussion that followed the first round of voting, the panel merged the two items describing neurological impairment and individual panellists expressed surprise at the low ranking of "necrotising enterocolitis" and "vascular thrombosis", leading to a discussion of these particular morbidities and their impact.

The results of the second round of voting are shown in table 2. While the panel's view of the top three morbidities (including the merged item of "New permanent neurological impairment (global or focal)) was clear, there was a large group of 21 candidates that could only be separated by applying tie-breaks. This group included "Necrotising enterocolitis" and "Vascular thrombosis". Given the lack of unambiguous group preference among these 21, the panel decided to request that the separate definition panel consider the 24 candidate morbidities given in bold in table 2, with the remainder discarded.

TABLE 2 HERE

For peer review only - http://bmjopen.bmj.com/site/about/guidelines.xhtml

Second panel meeting:

The initial assessment of the definition panel as to the feasibility of defining and monitoring each candidate morbidity still in contention after the first selection panel meeting is shown to the right of figure 2, and as presented to the panel in appendix 4.

Eleven candidate morbidities were considered straightforward to define and monitor in routine practice: “unplanned reoperation / reintervention”, “length of ICU stay”, “Major adverse event (eg cardiac arrest, ECMO, Serious Untoward Incident)”, “ECMO / mechanical support”, “necrotising enterocolitis”, “prolonged hospital length of stay”, “acute kidney injury”, “prolonged pleural effusion”, “vascular thrombosis”, “surgical bleeding” and “complete heart block”.

Six candidate morbidities were considered less straightforward: “new permanent neurological impairment (global or focal)”, “problems feeding”, “hospital acquired infection”, “poor communication between clinical team and family”, “complications during surgery”, “phrenic nerve injury”.

The remaining 7 candidate morbidities were deemed difficult to define and monitor in routine practice: “new impaired cognitive function more than a month after surgery”, “developmental delay”, “low cardiac output”, “mental health consequences”, “recurrent laryngeal nerve palsy”, “questionable clinical team decision & diagnosis”, and “level of support at home”.

The output from the causal mapping exercises given at Appendix 5 highlighted how “mental health consequences” could be a result of several other candidate morbidities, how the majority of candidate morbidities could result in prolonged stay in intensive care or hospital and how “low cardiac output” could become manifest in several of the other candidate morbidities.

Based on these assessments and the selection panel’s own previous assessment of the importance of these morbidities, it was agreed that the following candidate morbidities would be selected without further discussion: “New permanent neurological impairment (global or focal)”, “unplanned reoperation / reintervention”, “problems feeding”, “acute kidney injury” and “poor communication between clinical team and family”.

The following candidate morbidities were discarded at this point without further discussion: “mental health consequences”, “prolonged hospital stay”, “questionable clinical team decision and diagnosis” and “level of support at home”.

The remaining 15 candidate morbidities were then discussed, with the panel reminded of the need to select at most 5 from these 15. Group decisions were made to select “ECMO”, “major adverse event”, “necrotising enterocolitis”, “hospital acquired infection” and “recurrent laryngeal nerve palsy”. Note that the group accepted that “Major adverse event” should not include ECMO as this had been selected as a distinct morbidity.

Group decisions were made to discard at this stage “new impaired cognitive function more than one month after surgery”, “developmental delay”, “low cardiac output”, “prolonged

For peer review only - http://bmjopen.bmj.com/site/about/guidelines.xhtml

pleural effusion", "vascular thrombosis", "surgical bleeding", "complications during surgery", "phrenic nerve injury" and "length of ICU stay".

The online poll conducted after the second panel meeting identified "Recurrent laryngeal nerve palsy", "Phrenic nerve injury", "Complete heart block" and "prolonged pleural effusion" as potential substitute morbidities in the event of the definition panel vetoing any of the selected morbidities in routine practice.

Final rulings of definitions panel and chief investigators

Of the ten morbidities selected by the panel, 2 ("poor communication between clinical team and family" and "recurrent laryngeal nerve palsy") were removed from the final list by the definitions panel following input from and consultation with the chief investigators (VT and KB) and other members of the project management team. In each case this was done on the grounds that the morbidity concerned would be too problematic to measure in routine practice. The morbidity "prolonged pleural effusion" was added to the final list of morbidities as a substitute. Feeding problems due to symptomatic recurrent laryngeal nerve palsy were then included under "problems feeding". The definition panel and the project team decided to monitor the incidence of phrenic nerve injury and complete heart block by using routinely collected cardiac audit data. These changes were shared with the selection panel.

The final nine morbidities chosen for inclusion in the study, using the revised labels used in working up final definitions are given in table 3.

TABLE 3 HERE

Alongside these nine, the study team committed to measuring poor communication between the clinical team and the family among the 800+ patients anticipated to enter the matched cohort phase of our study and to conducting secondary analyses to identify the impact of longer stays in intensive care above and beyond the impact of the selected morbidities. A detailed description of the definition process and the final definitions used is available at [31].

DISCUSSION

The morbidities selected to be measured in approximately 3600 patients over 18 months starting October 2015 cover a range of phenomena associated with paediatric cardiac surgery including indicators of organ damage such as acute kidney injury and necrotising enterocolitis, acute events such as cardiac arrest (included as a "major adverse event"), the necessity for major interventions in the intra-operative period such as ECMO, and problems feeding that, while not necessarily a priority during the intra-operative period, are considered to have a considerable impact on children and families in the months that follow.

At each stage of selection, the morbidity that was ranked of greatest importance by the panel was neurological impairment. This came as little surprise to the study investigators and vindicates the inclusion in our overarching programme of research of a parallel evaluation of the "Brief Developmental Assessment", which it is hoped will provide a tool that can be

For peer review only - http://bmjopen.bmj.com/site/about/guidelines.xhtml

deployed by nursing staff to identify patients that would benefit from referral to specialist neurological or other developmental services. However, it is fair to say that inclusion on the panel of family representatives and clinicians from outside the tertiary surgical centres brought other issues such as problems feeding and poor communication between clinical teams and families to greater prominence than if the panel had consisted solely of tertiary clinicians or if the study investigators had chosen the morbidities themselves.

We found that opening up the process of choosing the metrics by which services should monitor their performance to include the perspectives of patients and family representatives, which is in line with policy initiatives in England (32), brought challenges. Throughout our work, there was a tension between choosing a “clean” set of “clinical” measures that most closely matched the understanding of “surgical morbidity” among the tertiary clinicians on the panel and the inclusion of arguably murkier phenomena considered hugely important by families and those working in secondary care.

In particular, those working in surgical centres were more concerned than family representatives and others with the attribution of morbidity to the surgical act, keen to include morbidities that may be related in part to surgical technique (laryngeal nerve palsy and phrenic nerve injury) and degree of success (low cardiac output) and anxious to avoid the attribution to surgical teams of morbidities that are currently considered to “come with the territory” of congenital heart disease and its surgical treatment. Family representatives and others highlighted the value of gathering information on the incidence and impact of key morbidities, even if they were not caused by surgery, not least as some of them may be reducible through interventions at other points in the care pathway.

We consider that features of our study design were key to drawing out and balancing these perspectives. The Nominal Group Technique, starting as it does with an opportunity for each panellist to speak without interruption and within an embedded democratic process, is specifically designed to minimise the influence of perceived power differentials and dominant personalities within a group. This was reinforced by the use of a secret ballot process to determine group preferences, allowing panellists to record their disagreement with the positions stated by others without that being openly declared. The voting tool used distinguishes between unambiguous group preferences and those that rely on tie-breaking. This acceptance and presentation of lack of consensus helped to focus discussion on where it would be most valuable to the task of selecting a group of morbidities and divert unnecessary debate focused on achieving a false consensus through attrition.

Also, our choice to separate the process of identifying which morbidities are most important from the process of assessing the feasibility of defining and monitoring morbidities prevented all parties from self-censoring and clinical panellists from unconsciously using or claiming privileged knowledge of measurement processes to strengthen the case for morbidities they wanted to include.

Firm and expert chairing was essential to maintaining this discipline. Having a chair conversant with the clinical area but also experienced in working with multi-stakeholder groups including patient and family representatives was also key. Although it meant that the Chair brought their own clinical perspective to the table, the panel benefitted from the Chair’s ability to discern between the wheat and chaff of clinical discussions and summarise for non-

For peer review only - http://bmjopen.bmj.com/site/about/guidelines.xhtml

clinical participants. It is also questionable whether a Chair that was not an accomplished surgeon and clinical researcher would have held the respect of all parties through the process.

Separating the processes of judging the importance and the feasibility of routinely monitoring morbidities did however risk some of the subtlety of discussions slipping through the gaps between two panels of people. Whilst the preparation of detailed summaries of panel meetings and the presence of the same facilitating team (CP and MU) at all meetings reduced this risk, we acknowledge that the defined morbidities that will be monitored do not correspond exactly in all cases to the phenomena deemed important by the selection panel.

In summary, we found that the inclusion of patient and family perspectives in identifying metrics for use in monitoring a highly specialised clinical service is a challenging but feasible exercise that can add valuable breadth to notions of quality and how to measure it.

CONTRIBUTORSHIP STATEMENT

CP helped design the study, organised and facilitated the panels, contributed to analysis, contributed to the first draft of the paper and approved the final draft. KB helped design the study as co-chief investigator, contributed to the drafting of the paper and approved the final draft. IM contributed to the causal mapping exercise and approved the final draft. HJ contributed to the causal mapping exercise and approved the final draft. JW helped design the study, contributed to the drafting of the paper and approved the final draft. LC reviewed literature for the selection panel and approved the final draft. AM chaired the definition panel that fed into the work and approved the final draft. TT chaired the selection panel, contributed to the drafting of the manuscript and approved the final draft. VT helped design the study as co-chief investigator, contributed to the panel and approved the final draft. MU helped design the study, facilitated the panels, conducted the causal mapping exercise, contributed to analysis, wrote the first draft of the paper and approved the final draft.

COMPETING INTERESTS

MU and CP are part of the team that developed the online voting tool CRANKIT used in this study.

FUNDING

This project was funded by the National Institute for Health Research
Health Services and Delivery Research Programme (project number 12/5005/06).

MU was in part supported by the National Institute for Health Research (NIHR) Collaboration for Leadership in Applied Health Research and Care (CLAHRC) North Thames at Bart's Health NHS Trust.

KB, JW and VT were supported by the National Institute for Health Research Biomedical Research Centre at Great Ormond Street Hospital for Children NHS Foundation Trust and University College London.

For peer review only - http://bmjopen.bmj.com/site/about/guidelines.xhtml

For peer review only

The views and opinions expressed are those of the authors and do not necessarily reflect those of the Health Services and Delivery Research Programme, NIHR, NHS or the Department of Health.

DATA SHARING AGREEMENT

There are no additional data available.

ACKNOWLEDGEMENTS

We would like to thank Rehana Ahmed, David Barron, Jane Cassidy, Kate English, Allan Goldman, Samantha Johnson, Ravi Kumar, Andrew Parry, Samana Schwank, Emma Simmonds, Rob Tulloh, Emma Twigg. Rehana Ahmed, David Barron, Jane Cassidy, Kate English, Allan Goldman, Samantha Johnson, Ravi Kumar, Andrew Parry, Samana Schwank, Emma Simmonds, Rob Tulloh, and Emma Twigg for their contribution to this work.

REFERENCES

1. Aylin P, Alves B, Best N, Cook A, Elliott P, Evans SJ, et al. Comparison of UK paediatric cardiac surgical performance by analysis of routinely collected data 1984–96: was Bristol an outlier? The Lancet. 2001 Jul;358(9277):181–7.

2. Crowe S, Brown KL, Pagel C, Muthialu N, Cunningham D, Gibbs J, et al. Development of a diagnosis- and procedure-based risk model for 30-day outcome after pediatric cardiac surgery. J Thorac Cardiovasc Surg. 2013 May;145(5):1270–8.

3. Jacobs JP, O'Brien SM, Pasquali SK, Jacobs ML, Lacour-Gayet FG, Tchervenkov CI, et al. Variation in outcomes for risk-stratified pediatric cardiac surgical operations: an analysis of the STS Congenital Heart Surgery Database. Ann Thorac Surg. 2012 Aug;94(2):564-571-572.

4. Jenkins KJ, Gauvreau K, Newburger JW, Spray TL, Moller JH, Iezzoni LI. Consensus-based method for risk adjustment for surgery for congenital heart disease. J Thorac Cardiovasc Surg. 2002 Jan;123(1):110–8.

5. Kang N, Cole T, Tsang V, Elliott M, de Leval M. Risk stratification in paediatric open-heart surgery. Eur J Cardio-Thorac Surg Off J Eur Assoc Cardio-Thorac Surg. 2004 Jul;26(1):3–11.

6. Kang N, Tsang VT, Gallivan S, Sherlaw-Johnson C, Cole TJ, Elliott MJ, et al. Quality assurance in congenital heart surgery. Eur J Cardio-Thorac Surg Off J Eur Assoc Cardio-Thorac Surg. 2006 May;29(5):693-697-698.

7. Lacour-Gayet F, Clarke D, Jacobs J, Gaynor W, Hamilton L, Jacobs M, et al. The Aristotle score for congenital heart surgery. Semin Thorac Cardiovasc Surg Pediatr Card Surg Annu. 2004;7(1):185–91.

8. O'Brien SM, Clarke DR, Jacobs JP, Jacobs ML, Lacour-Gayet FG, Pizarro C, et al. An empirically based tool for analyzing mortality associated with congenital heart surgery. J Thorac Cardiovasc Surg. 2009 Nov;138(5):1139–53.

For peer review only - http://bmjopen.bmj.com/site/about/guidelines.xhtml

9. Pagel C, Brown KL, Crowe S, Utley M, Cunningham D, Tsang V. A mortality risk model to adjust for case mix in UK paediatric cardiac surgery. Health Serv Deliv Res. 2013 May;1(1).

10. Pagel C, Utley M, Crowe S, Witter T, Anderson D, Samson R, et al. Real time monitoring of risk-adjusted paediatric cardiac surgery outcomes using variable life-adjusted display: implementation in three UK centres. Heart Br Card Soc. 2013 Apr 5;

11. Cunningham D, Franklin R, Bridgewater B, Deanfield JE. Investigation of mortality from Paediatric Cardiac Surgery in England 2009-12: 8 April 2013 [Internet]. 2013 [cited 2013 Aug 17]. Available from: http://www.ucl.ac.uk/nicor/paediatric_cardiac_surgery_report

12. Jacobs ML, Jacobs JP, Franklin RCG, Mavroudis C, Lacour-Gayet F, Tchervenkov CI, et al. Databases for assessing the outcomes of the treatment of patients with congenital and paediatric cardiac disease--the perspective of cardiac surgery. Cardiol Young. 2008 Dec;18 Suppl 2:101–15.

13. Jenkins KJ, Beekman Iii RH, Bergersen LJ, Everett AD, Forbes TJ, Franklin RCG, et al. Databases for assessing the outcomes of the treatment of patients with congenital and paediatric cardiac disease--the perspective of cardiology. Cardiol Young. 2008 Dec;18 Suppl 2:116–23.

14. Shahian DM, Edwards FH, Jacobs JP, Prager RL, Normand S-LT, Shewan CM, et al. Public Reporting of Cardiac Surgery Performance: Part 1--History, Rationale, Consequences. Ann Thorac Surg. 2011 Sep 1;92(3_Supplement):S2-11.

15. Boseley S, editor health. Leeds General Infirmary to resume children's heart surgery. The Guardian [Internet]. 2013 Apr 8 [cited 2014 Mar 20]; Available from: http://www.theguardian.com/society/2013/apr/08/leeds-general-childrens-heart-surgery

16. Bower H. London hospital orders inquiry into paediatric heart surgery deaths. BMJ. 1999 Aug 7;319(7206):335–335.

17. Dyer C. Consultation over children's heart surgery was unfair to Royal Brompton, judge rules. BMJ. 2011 Nov 8;343(nov08 2):d7275–d7275.

18. Mayor S. Oxford hospital suspends paediatric cardiac surgery after four deaths. BMJ. 2010 Mar 4;340(mar04 2):c1303–c1303.

19. McGauran A. Fewer hospitals in England should perform children's heart surgery. BMJ. 2011 Feb 18;342(feb18 1):d1129–d1129.

20. Murphy JF. The Bristol Royal Infirmary Inquiry 18th July 2001. Ir Med J. 2001 Sep;94(8):228.

21. Brown KL, Crowe S, Franklin R, McLean A, Cunningham D, Barron D, et al. Trends in 30-day mortality rate and case mix for paediatric cardiac surgery in the UK between 2000 and 2010. Open Heart. 2015;2(1):e000157.

22. Belliveau D, Burton HJ, O'Blenes SB, Warren AE, Hancock Friesen CL. Real-time complication monitoring in pediatric cardiac surgery. Ann Thorac Surg. 2012 Nov;94(5):1596–602.

For peer review only

For peer review only - http://bmjopen.bmj.com/site/about/guidelines.xhtml

23. Cooke M, Thackray S. Differences between community professional and patient perceptions of chronic obstructive pulmonary disease treatment outcomes: a qualitative study. J Clin Nurs. 2012 Jun;21(11–12):1524–33.

24. NIHR Evaluation, Trials and Studies | 12/5005/06 [Internet]. [cited 2014 Apr 3]. Available from: http://www.nets.nihr.ac.uk/projects/hsdr/12500506

25. Gallagher M, Hares T, Spencer J, Bradshaw C, Webb I. The Nominal Group Technique: A Research Tool for General Practice? Fam Pract. 1993 Mar 1;10(1):76–81.

26. Jones J, Hunter D. Consensus methods for medical and health services research. BMJ. 1995 Aug 5;311(7001):376–80.

27. Mullen PM. Delphi: myths and reality. J Health Organ Manag. 2003;17(1):37–52.

28. Keeney S, Hasson F, McKenna HP. A critical review of the Delphi technique as a research methodology for nursing. Int J Nurs Stud. 2001 Apr;38(2):195–200.

29. Utley M, Gallivan S, Mills M, Mason M, Hargraves C. A consensus process for identifying a prioritised list of study questions. Health Care Manag Sci. 2007 Feb;10(1):105–10.

30. HS&DR - 12/5005/06: Selection, definition and evaluation of important early morbidities associated with paediatric cardiac surgery [Internet]. [cited 2014 Nov 4]. Available from: http://www.nets.nihr.ac.uk/projects/hsdr/12500506

31 Brown KL, Pagel C, Brimmell R, Bull K, Davis P, Franklin RC, Hoskote A, Khan N, Rodrigues W, Thorne S, Smith L, Chigaru L, Utley M, Wray J, Tsang V, Mclean A, Definition of important early morbidities related to paediatric cardiac surgery, Cardiology in the Young. 2016 Sep 29:1-10.

32. NHS England » Patient Centred Outcome Measures [Internet]. [cited 2016 Jan 3]. Available from: https://www.england.nhs.uk/ourwork/pe/pcoms/

For peer review only

For peer review only - http://bmjopen.bmj.com/site/about/guidelines.xhtml

FIGURES AND TABLES

Figure 1: the role of the selection panel within our wider study to identify and measure the incidence and impact of important morbidities following paediatric cardiac surgery

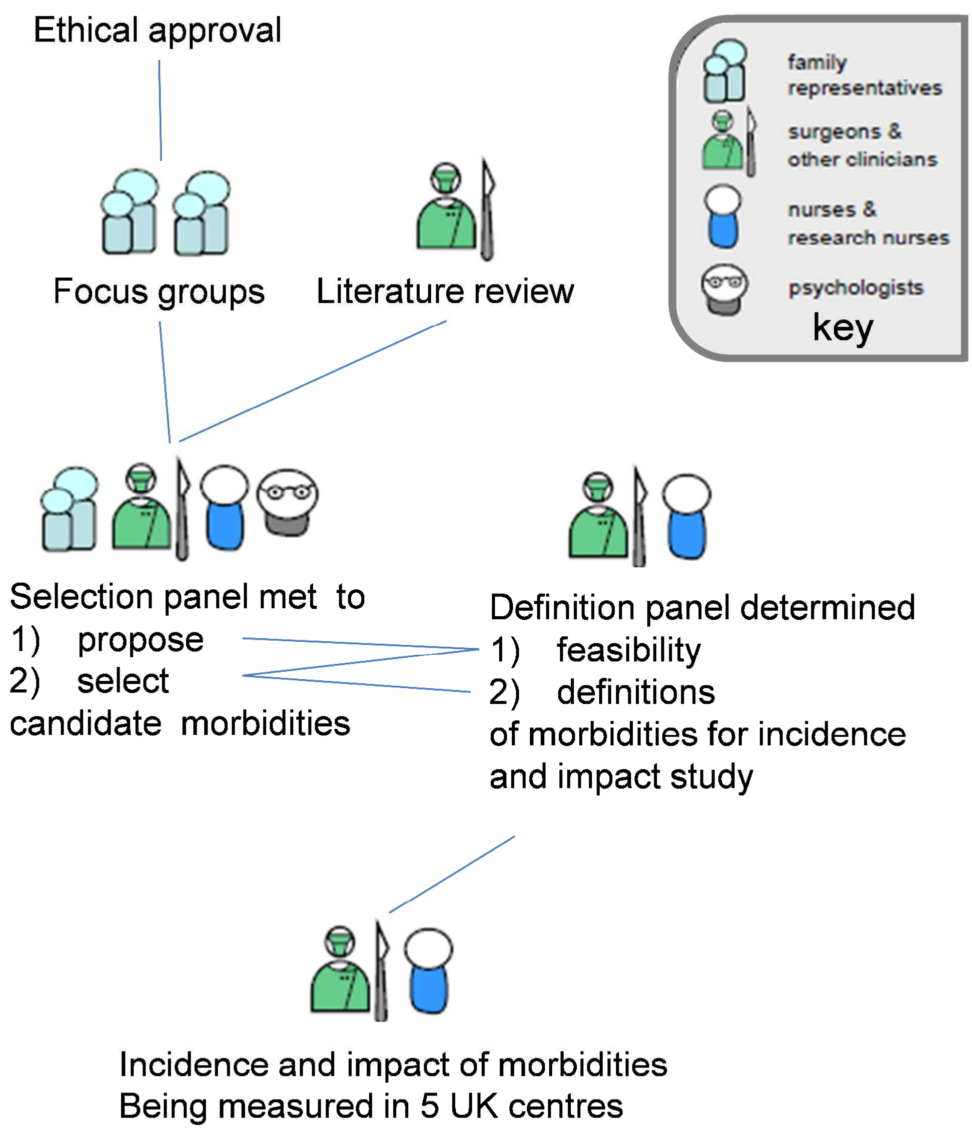

For peer review only - http://bmjopen.bmj.com/site/about/guidelines.xhtml

Figure 2 - Overview of results of selection process

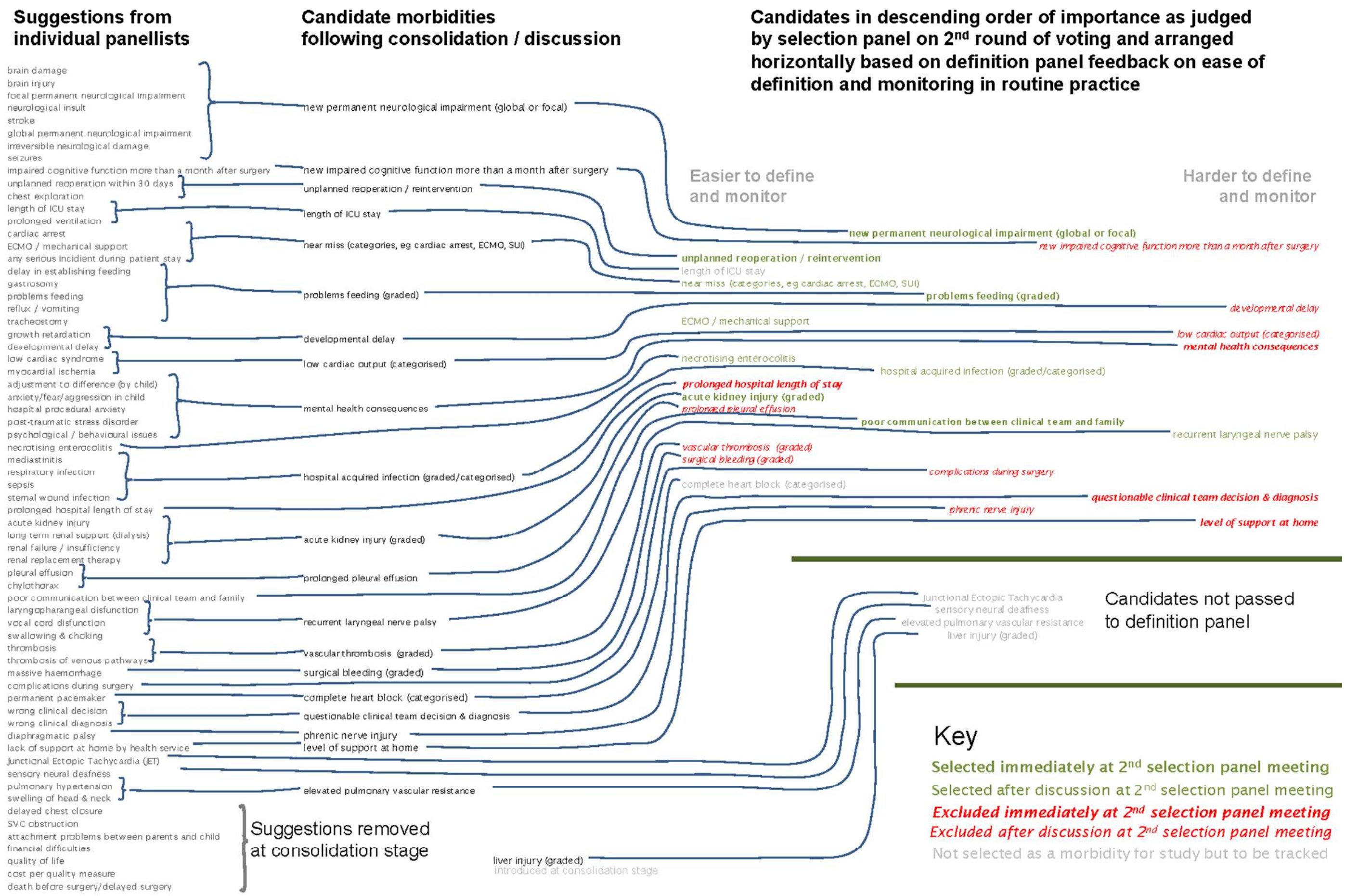

Table 1- results of the first ranking exercise

**Morbidities**	Rank before tie-breaks	Rank after tie-breaks
New global permanent neurological impairment	1	1
New impaired cognitive function more than a month after surgery	2	2
Unplanned reoperation / reintervention	2	3
Developmental delay	2	4
Major adverse event	2	5
Problems feeding (graded)	2	6
Mental health consequences	2	7
Length of ICU stay	2	8
New focal permanent neurological impairment	2	9
Low cardiac output (categorised)	2	10
Poor communication between clinical team and family	2	11
ECMO / mechanical support	2	12
Acute kidney injury (graded)	2	13
Prolonged pleural effusion	2	14
Complications during surgery	2	15
Hospital acquired infection (graded/categorised)	2	15
Prolonged hospital length of stay	2	17
Complete heart block (categorised)	2	18
Surgical bleeding (graded)	2	19
Questionable clinical team decision & diagnosis	2	20
Level of support from hospital available at home	2	21
Recurrent laryngeal nerve palsy	2	21
Phrenic nerve injury	2	23
Necrotising enterocolitis	24	24
Vascular thrombosis (graded)	25	25
JET	26	26
Elevated pulmonary vascular resistance	27	27
Liver injury (graded)	27	27
Sensory neural deafness	27	27

For peer review only - http://bmjopen.bmj.com/site/about/guidelines.xhtml

Table 2 – Results of the second round of ranking. The morbidities passed to the definition panel for consideration are shown in **bold**.

Morbidities	Rank before tie-breaks	Rank after tie-breaks
**New permanent neurological impairment (global or focal)**	1	1
**New impaired cognitive function more than a month after surgery**	2	2
**Unplanned reoperation / reintervention (categorisation)**	3	3
**Length of ICU stay**	4	4
**Major adverse event**	4	5
**Problems feeding (graded)**	4	6
**Developmental delay**	4	7
**ECMO / mechanical support**	4	8
**Low cardiac output (categorised)**	4	8
**Mental health consequences**	4	10
**Necrotising enterocolitis**	4	11
**Hospital acquired infection (graded/categorised)**	4	12
**Prolonged hospital length of stay**	4	13
**Acute kidney injury (graded)**	4	14
**Prolonged pleural effusion**	4	15
**Poor communication between clinical team and family**	4	16
**Recurrent laryngeal nerve palsy**	4	17
**Vascular thrombosis (graded)**	4	18
**Surgical bleeding (graded)**	4	19
**Complications during surgery**	4	20
**Complete heart block (categorised)**	4	21
**Questionable clinical team decision & diagnosis**	4	22
**Phrenic nerve injury**	4	23
**Level of support at home**	4	24
JET	25	25
Sensory neural deafness	26	26
Elevated pulmonary vascular resistance	27	27
Liver injury (graded)	27	27

For peer review only - http://bmjopen.bmj.com/site/about/guidelines.xhtml

Table 3 – The final list of 9 selected morbidities

Morbidities
Acute neurological event
Unplanned reoperation / re-intervention
Problems feeding (excluding NEC)
Need for renal replacement therapy (excluding ECMO)
Major adverse event
ECLS / ECMO
Necrotising enterocolitis (NEC)
Hospital acquired infection (graded/categorised)
Prolonged pleural effusion / chylothorax

For peer review only

Appendix 1

Voting process used in selection

The voting process used as part of the nominal group technique deployed was based on the algorithm presented in *Utley M, Gallivan S, Mills M, Mason M, Hargraves C. A consensus process for identifying a prioritised list of study questions. Health Care Manag Sci. 2007 Feb;10(1):105–10*. A short summary is presented here.

In a secret ballot, each panellist ranked the candidate options in order of descending importance. They were permitted to use tied ranks and were not obliged to rank every option. For each and every possible pair of options (candidate morbidities A and B say) we determined whether at least as many participants preferred option A to option B as preferred option B to option A. We then used the analysis summarised in Box 1 to identify group preferences among the set of candidate morbidities.

In the first panel meeting, the rankings supplied by individual panellists were entered into an Excel spreadsheet and a VBA Macro written by MU and CP was used to conduct the analysis. For the online poll conducted after the second panel meeting, we used the web-based tool available at www.crankit.io.

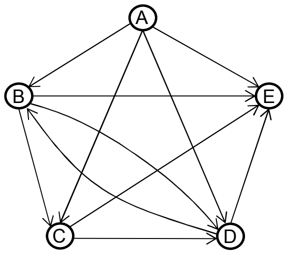

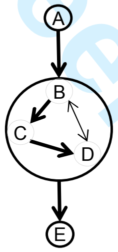

Rank before tie-breaks	Option	Rank after tie-breaks
1	A	1
2	B	2
2	C	3
2	D	4
5	E	5

Step 1

Draw an arrow from option A to B if at least as many participants ranked A higher than B as ranked B higher than A. Repeat process in each direction for all pairs of options.

In this example there is an arrow in both directions between B and D, indicating that exactly as many participants preferred B to D as preferred D to B…

Step 2

Identify the hierarchy of subsets of options. Within a subset there is no clear group ranking of options.

…there is a majority preference for A above every other option and E is least favoured. The cycle of arrows between B, C and D indicates that there is no unambiguous ranking of these 3...

Step 3

Try to rank options within subsets by first counting the arrows leaving each option within that subset and then, if there are ties, counting just those arrows between tied options.

… there are two arrows leaving B and just 1 leaving each of C and D, so the first tie-break places B above C and D. Then, considering just C and D, the second tie break places C above D.

Box 1; explanation of the robust group ranking process used in the nominal group approach employed by the selection panel.

For peer review only

Appendix 2

Panel composition

Name	Organisation	Role on panel
Ms Rehana Ahmed*	Independent	Family representative
Mr David Barron	Birmingham Children's Hospital	Heart surgeon
Dr Jane Cassidy†	Birmingham Children's Hospital	Intensive care doctor
Dr Kate English†	Leeds General Infirmary	Cardiologist
Dr Allan Goldman	Great Ormond Street Hospital	Intensive care doctor
Dr Helen Jepps†	Bradford Royal Infirmary	Paediatrician
Ms Samantha Johnson	Children's Heart Federation	Family representative
Dr Ravi Kumar*	Royal Berkshire Hospital	Paediatrician
Ms Isobel Macleod	Royal Hospital for Sick Children, Glasgow	Intensive care nurse
Mr Andrew Parry*	University Hospitals Bristol	Heart surgeon
Dr Samana Schwank	Children's Heart Federation	Family representative
Ms Emma Simmonds†	Evelina London Children's Hospital	Clinical nurse specialist
Professor Tom Treasure	Independent	Chairperson
Mr Victor Tsang	Great Ormond Street Hospital	Heart surgeon
Professor Rob Tulloh†	University Hospitals Bristol	Cardiologist
Dr Emma Twigg†	Great Ormond Street Hospital	Clinical psychologist

Table A2 Members of the selection panel. * indicates members who could not attend the 1st panel meeting, † those who could not attend the 2nd meeting. Members are listed in alphabetical order.

For peer review only - http://bmjopen.bmj.com/site/about/guidelines.xhtml

Appendix 3: The initial 66 terms under consideration in selection panel 1.

anxiety/fear/aggression	prolonged ventilation
problems feeding	complications during surgery
reflux/vomiting	developmental delay
pleural effusion	financial difficulties
swallowing & choking	lack of support at home
global permanent neurological impairment	laryngopharyngeal dysfunction
Mediastinitis	vocal cord dysfunction
myocardial ischemia	delay in establishing feeding
focal permanent neurological impairment	brain damage
sensory neural deafness	low cardiac output
brain injury	Sepsis
cardiac arrest	gastrosomy
renal failure/insufficiency	massive haemorrhage
length of ICU stay	thrombosis
post-traumatic stress disorder	impaired cognitive function more than a month after surgery
ECMO / mechanical support	quality of life
communication	prolonged hospital length of stay
hospital procedural anxiety	permanent pacemaker
adjustment to difference	long term renal support
Attachment	any serious incident during patient stay
neurological insult	cost per quality measure
Chylothorax	seizures
acute kidney injury	stroke
necrotising enterocolitis	sternal wound infection
junctional ectopic tachycardia (JET)	psychological/behavioural issues
pulmonary hypertension	growth retardation
SVC obstruction	tracheostomy
swelling of head & neck	diaphragmatic palsy
chest exploration	delayed chest closure
thrombosis of venous pathways	respiratory infection
irreversible neurological damage	wrong clinical decision
renal replacement therapy	wrong clinical diagnosis
unplanned reoperation within 30 days	death before surgery/delayed surgery

For peer review only - http://bmjopen.bmj.com/site/about/guidelines.xhtml

Appendix 4 - The summary of the feasibility of defining and measuring in routine practice (as judged by the definition panel) vs importance shown to the selection panel at their second meeting.

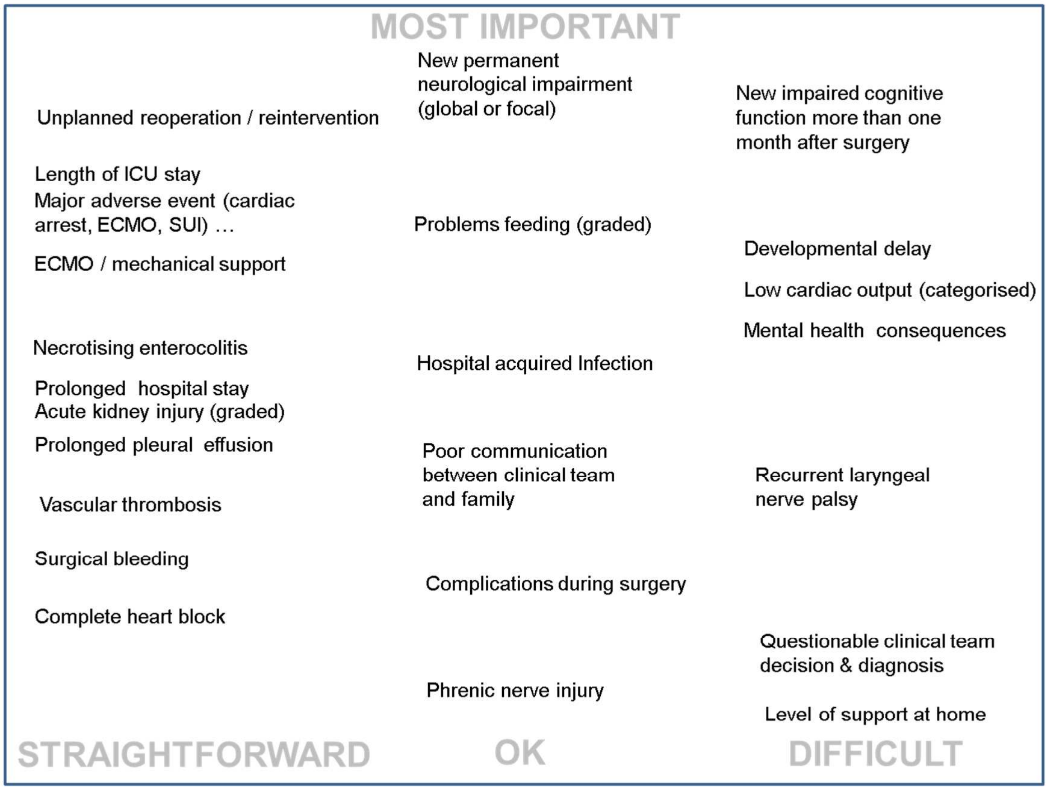

Appendix 5

Graphics shown to the selection panel at the second meeting on the relationships between candidate morbidities (figures A1-A3).

Figure A1

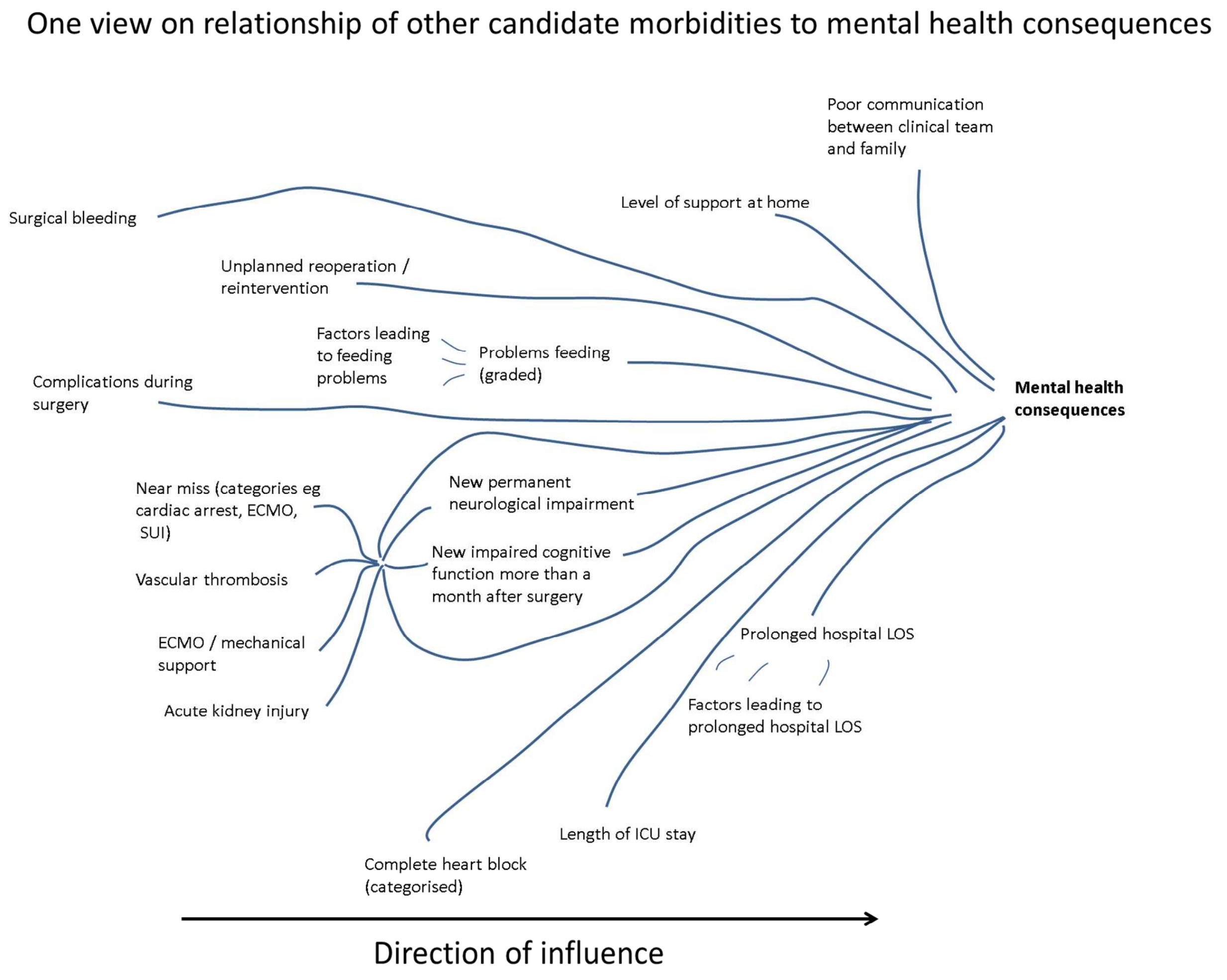

Figure A2

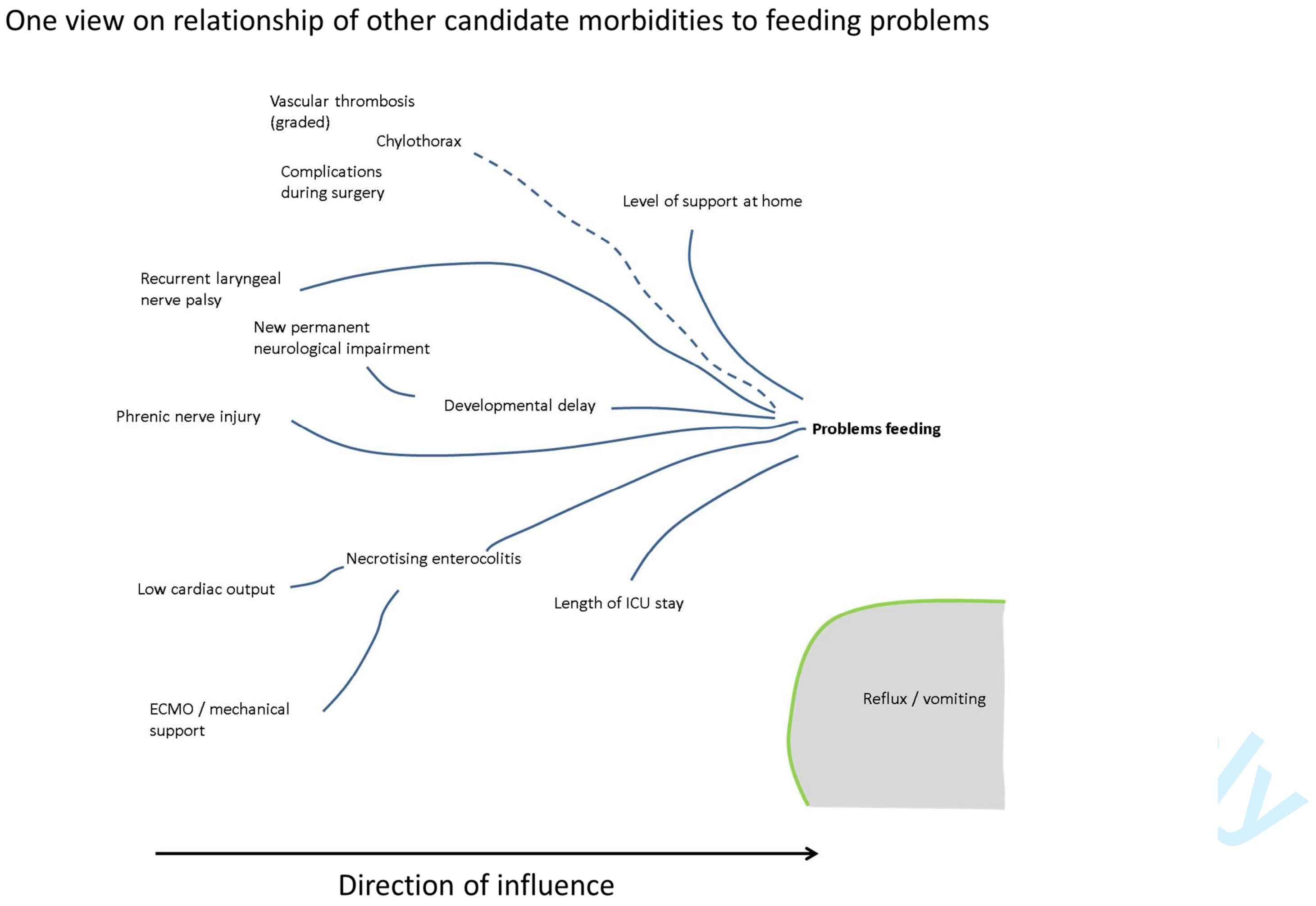
One view on relationship of other candidate morbidities to feeding problems
Vascular thrombosis (graded)
Chylothorax
Complications during surgery
Level of support at home
Recurrent laryngeal nerve palsy
New permanent neurological impairment
Developmental delay
Phrenic nerve injury
Problems feeding
Necrotising enterocolitis
Low cardiac output
Length of ICU stay
ECMO / mechanical support
Reflux / vomiting
Direction of influence

Figure A3

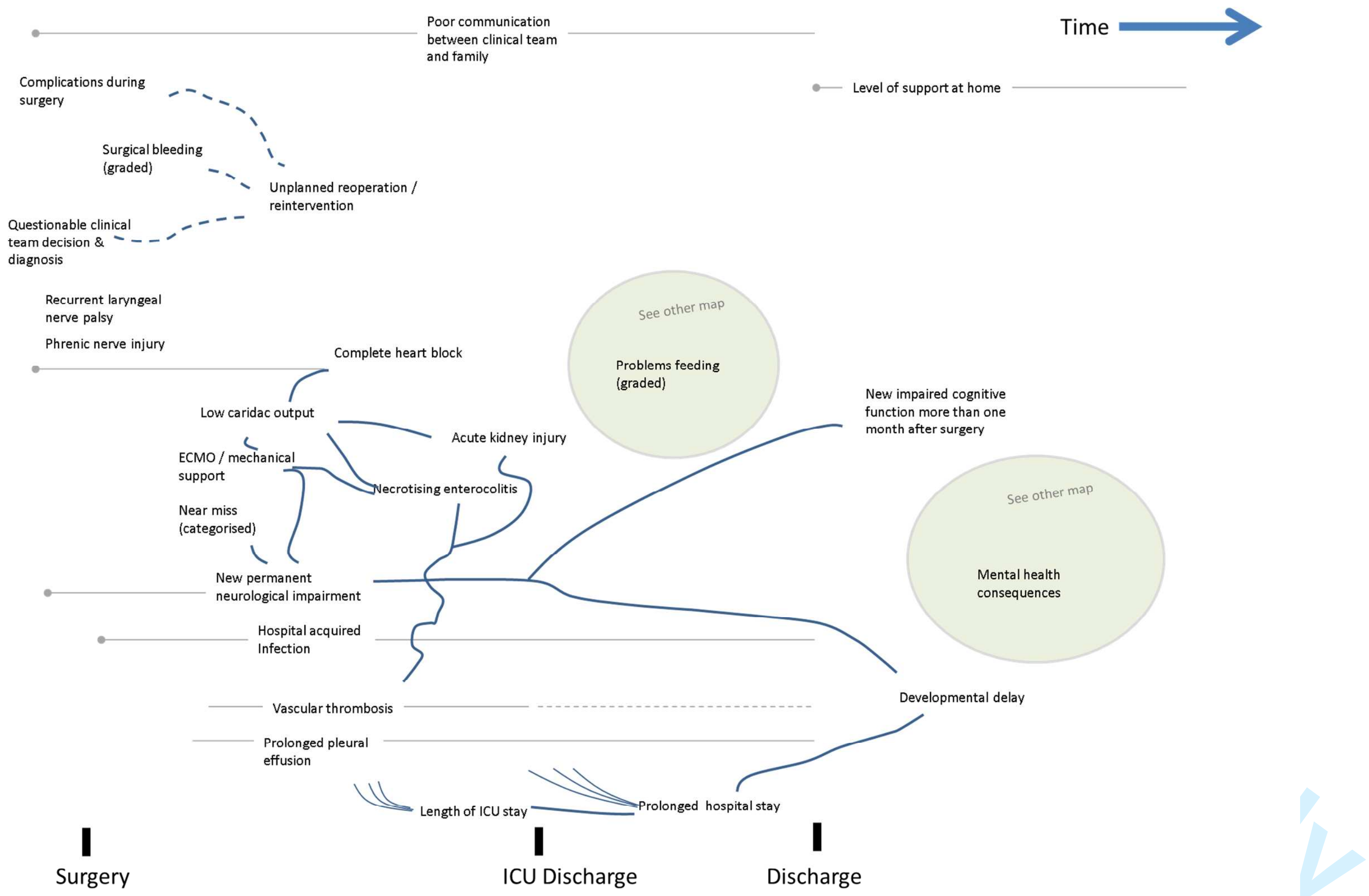

One view of whole picture (relationships of candidate morbidities to each other)

BMJ Open

Selection by a panel of clinicians and family representatives of important early morbidities associated with paediatric cardiac surgery suitable for routine monitoring using the nominal group technique and a robust voting process.

Journal:	*BMJ Open*
Manuscript ID	bmjopen-2016-014743.R1
Article Type:	Research
Date Submitted by the Author:	09-Jan-2017

Complete List of Authors:	Pagel, Christina; UCL, Clinical Operational Research Unit<br>Brown, Katherine; Great Ormond Street Hospital for Children, Cardiorespiratory Division<br>Macleod, Isobel; Royal Hospital for Children<br>Jepps, Helen; Bradford Teaching Hospitals NHS Foundation Trust<br>Wray, Jo; Great Ormond Street Hospital for Children, Cardiorespiratory Division<br>Chigaru, Linda; Great Ormond Street Hospital for Children, Cardiorespiratory Division<br>Mclean, Andrew; Royal Hospital for Children<br>Treasure, Tom; UCL, CORU Mathematics<br>Tsang, Victor; Great Ormond Street Hospital for Children, Cardiorespiratory Division<br>Utley, Martin; University College London, Clinical Operational Research Unit, Department of Mathematics

**Primary Subject Heading**:	Surgery
Secondary Subject Heading:	Patient-centred medicine, Research methods, Health services research
Keywords:	Paediatric cardiac surgery < PAEDIATRIC SURGERY, AUDIT, Quality in health care < HEALTH SERVICES ADMINISTRATION & MANAGEMENT

SCHOLARONE™ Manuscripts

Selection by a panel of clinicians and family representatives of important early morbidities associated with paediatric cardiac surgery suitable for routine monitoring using the nominal group technique and a robust voting process.

Authors: Christina Pagel, Katherine L Brown, Isobel McLeod, Helen Jepps, Jo Wray, Linda Chigaru, Andrew McLean, Tom Treasure, Victor Tsang, Martin Utley

Contributors: Rehana Ahmed, David Barron, Jane Cassidy, Kate English, Allan Goldman, Samantha Johnson, Ravi Kumar, Andrew Parry, Samana Schwank, Emma Simmonds, Rob Tulloh, Emma Twigg.

Email address for corresponding author: m.utley@ucl.ac.uk

Abstract

Objective: With survival following paediatric cardiac surgery improving, the attention of quality assurance and improvement initiatives is shifting to long-term outcomes and early surgical morbidities. We wanted to involve family representatives and a range of clinicians in selecting the morbidities to be measured in a major UK study.

Setting: Paediatric cardiac surgery services in the UK.

Participants: We convened a panel comprising family representatives, paediatricians from referring centres and surgeons and other clinicians from surgical centres.

Primary and secondary outcome measures: Using the Nominal Group Technique augmented by a robust voting process to identify group preferences, suggestions for candidate morbidities were elicited, discussed, ranked and then shortlisted. The shortlist was passed to a clinical group that provided a view on the feasibility of monitoring each shortlisted morbidity in routine practice. The panel then met again to select a prioritised list of morbidities for further study, with the list finalised by the clinical group and chief investigators.

Results: At the first panel meeting, 66 initial suggestions were made, with this reduced to a shortlist of 24 after two rounds of discussion, consolidation and voting. At the second meeting, this shortlist was reduced to 10 candidate morbidities. Two were dropped on grounds of feasibility and replaced by another the panel considered important. The final list of 9 morbidities included indicators of organ damage, acute events and feeding problems. Family representatives and clinicians from outside tertiary centres brought some issues to greater prominence than if the panel had consisted solely of tertiary clinicians or study investigators.

Conclusion: The inclusion of patient and family perspectives in identifying metrics for use in monitoring a specialised clinical service is challenging but feasible and can broaden notions of quality and how to measure it.

Word counts: Abstract: 287 Main text**:** 3884.

For peer review only - http://bmjopen.bmj.com/site/about/guidelines.xhtml

Strengths and limitations

The Nominal Group Technique, augmented by a robust secret voting process, allowed us to incorporate the perspectives of family representatives and clinicians from different professional groups in selecting early surgical morbidities they felt important to monitor in routine practice.

The robust voting process used identified group preferences from the preferences expressed by individual panellists and identified where there was a lack of consensus, guiding further discussion.

One limitation of the approach adopted was that it reflied on firm and expert chairing.

There was some unresolved tension between selecting morbidities clearly attributable to the surgical act and morbidities that are important to families but can be considered to "come with the territory" of congenital heart disease and its management.

Relying on a face-to-face approach necessarily limited the size of the panel and we cannot claim the priorities and preferences expressed are representative of the respective professional groups and of families in general.

Introduction

Early mortality following paediatric cardiac surgery, defined as death within 30 days of surgery or death prior to discharge home, has been the focus of many research studies (1–10), audit initiatives (6,11–14) and, particularly in the UK, of public scrutiny (1,15–20) over recent decades. However, with early mortality having fallen to 2-3% (21), attention has shifted to longer term outcomes and to broadening the assessment of early outcomes to include early morbidities.

Reporting of early morbidities associated with paediatric cardiac surgery has been driven largely by the data available within the databases of professional societies (12) or those available within datasets curated by individual clinical teams (22). Quality assurance initiatives rooted in such datasets benefit greatly from considerable effort, often over many years, to agree definitions of the outcomes collected and design data collection processes. However, it is inevitable that the datasets agreed upon, constructed and curated by clinicians (as individuals or via professional societies) focus largely on outcomes considered important from the perspective of that clinician or professional group. Research in other specialties has shown that patients and carers can have quite different perceptions to clinicians on what outcomes are important to monitor as part of service evaluation (23).

We report here on a process used to select early morbidities as part of a study to identify and then measure the incidence and impact of important early morbidities among paediatric cardiac surgery patients (NIHR HS&DR 12/5005/06 (24)). A key aim of our work was to incorporate a broad set of perspectives, including those from family representatives and professionals from different sectors on what early morbidities were important to monitor in routine practice.

Methods

Overview

Figure 1 gives an overview of the role of our selection process within the wider study. A panel of clinicians and patient representatives met twice to shortlist and then to select early morbidities, the incidence and

For peer review only - http://bmjopen.bmj.com/site/about/guidelines.xhtml

impact of which they considered important to measure. As shown in Figure 1, our selection process was linked to a parallel process of defining potential morbidities. The shortlist of morbidities produced after the first meeting of the selection panel was considered by a separate group composed entirely of clinicians. This definition panel provided a view on the feasibility of defining, measuring and routinely monitoring each shortlisted morbidity, which informed the second selection meeting.

Figure 1 HERE

Composition of the selection panel

In forming the panel, we aimed to include clinicians from surgical centres, referring hospitals and primary care as well as family representatives. We wanted a panel of enough people to provide a range of perspectives while being manageable.

The panel comprised 15 people: 3 family representatives, 3 paediatric cardiac surgeons, 2 paediatric intensive care doctors, 2 paediatric cardiologists, 2 paediatricians, a paediatric intensive care nurse, a clinical nurse specialist and a clinical psychologist with experience of working with children with congenital heart disease and their families. The panel was chaired by a cardiothoracic surgeon with extensive experience of chairing multi-disciplinary panels. Two of the three family representatives were nominated by the Children's Heart Foundation, a parent-led charity and umbrella organisation of CHD charities and voluntary organisations. The third had facilitated one of the focus groups that fed into the selection process. We tried, but did not manage, to recruit a GP to the panel.

With the permission of panel members, both selection panel meetings were recorded and professionally transcribed. Each selection panel meeting also had a pre-determined seating plan to ensure that people from similar specialities were not grouped together.

Selection panel meeting 1: shortlisting

The aim of the first meeting was to identify a shortlist of 15-20 candidate morbidities that would then be considered by the definitions group. Prior to the meeting, the panel was supplied with an extensive list of candidate morbidities identified through:

- an ongoing systematic review conducted as part of our wider programme of research;
- three facilitated focus groups held in different UK cities with parents recruited by the Children's Heart Federation;
- an online forum for patients and families hosted on the website of the Children's Heart Federation.

The focus groups and literature review will be the subject of other publications. The panel was also sent an abridged version of the study protocol, a description of the role of the selection panel and an agenda. Each panellist was asked to identify among or beyond this list of candidate morbidities, those they judged most important to monitor routinely according to a deliberately broad working definition of a surgical morbidity as:

> *Any health or emotional problem that arose as a result of the fact of surgery (whether directly caused by surgery/post-operative care or not).*

For peer review only - http://bmjopen.bmj.com/site/about/guidelines.xhtml

For the first meeting, panellists were requested not to censor their suggestions on grounds of the perceived difficulty of definition or measurement and it was stressed that another group would be making these judgements.

We used the nominal group technique (25,26) augmented by a robust voting process to determine group rankings of morbidities. The nominal group technique is designed to reduce the influence of perceived power differentials and of dominant personalities on group decision making whilst retaining the benefit of discussion absent from other systematic approaches to group decision making such as Delphi (27,28). We inferred group preferences from individual rankings using a method developed by Utley et al (29) that is briefly described in appendix 1.

Structure of the first panel meeting

Each panellist was given the opportunity to speak uninterrupted for two minutes on the morbidities they considered important. Each suggestion was entered onto a spreadsheet which was projected in the meeting room to ensure accurate transcription. The panel was then given the opportunity to add to this initial list if they thought something important had been missed.

The Chair led a process of identifying suggestions that fell outside the working definition above, duplication among suggestions and merging of closely related suggestions. There was then a secret ballot in which panellists were asked individually to rank the resulting list of candidate suggestions in order of descending importance. The voting process and the method for generating group preferences from individual ranking data are described further in appendix 1.

During a scheduled break, the group preferences were calculated from the individual rankings by two of the authors (CP, MU) who did not have a vote on which morbidities to measure. After the break, the group preferences were fed back to the panel. The Chair then led a second round of discussion, focussing on the group preferences and giving panellists the opportunity to argue for specific morbidities being given greater importance, and for the group to further consolidate the list of morbidities.

There was a second round of secret ranking followed by feedback of group preferences prior to a consensus being sought as to the prioritised shortlist of 15 to 20 morbidities to be passed to the separate definitions panel for an assessment on the feasibility of defining, measuring and monitoring each in routine practice.

Causal mapping

Following the first selection group meeting, it was decided that it might be useful for non-clinical members of the panel to have an accessible summary of any causal relationships among candidate morbidities so that, when choosing the best set of morbidities to monitor, any overlap or redundancy among candidates could be accounted for. To this end, a set of causal mapping exercises was conducted by one of the facilitative team (MU) separately with two of the panellists (IM and HJ). In each exercise, cards representing shortlisted morbidities were placed on a large sheet of paper and arranged left to right with lines drawn to indicate potential causal relationships. Photographs were taken of these causal maps which were then converted to diagrams.

Selection panel meeting 2: incorporating feasibility and overlap.

Prior to the second meeting, panellists were provided with a pack of materials containing:

For peer review only - http://bmjopen.bmj.com/site/about/guidelines.xhtml

- a summary of any estimates of incidence and impact of candidate morbidities from the systematic review
- the judgment of the definition panel on the feasibility of defining, measuring and monitoring routinely each candidate morbidity
- summary of potential long term impacts of each candidate morbidity from the definition panel
- a summary of the parent and family focus groups and the online forum
- the diagrams generated through the causal mapping exercise.
- minutes from the first meeting
- a statement of the purpose of the second meeting and its agenda

At the beginning of the second meeting the panel were given a brief reminder of the scope of the overall project, the remit of the selection panel and the timescales we were working to.

The panel were tasked with narrowing the list of shortlisted candidates to a selection of 6-10, the incidence and impact of which would be measured in five centres over 18 months (30). It was explained that the upper limit of 10 morbidities was due to the sample size required for measuring the impact of distinct morbidities. It was explained that we could measure just the incidence of other morbidities if possible from routine data.

Panellists were also alerted to the (then) recently launched NHS England consultation on the future of Children's Heart Services in England, which highlighted the possibility of future national audit of surgical morbidities.

Panellists were asked to consider the following:

- *Feasibility of measurement, including timescales*. Given our plans to measure the impact of morbidities, the project team stressed that selected morbidities needed to be identifiable in a timely manner.
- *Overlap and redundancy among selected morbidities*. The project team made the point that selecting morbidities that almost always occur with other selected morbidities would pose problems in terms of measuring their individual impact. It highlighted that length of stay measures are particularly problematic in this respect.
- *Incidence*. Selected morbidities needed to have an incidence of at least 1.5-2% for us to measure their impact over 18 months due to sample-size considerations.

A summary of the judgements of the definition panel was presented to the panel, consisting of an array showing the shortlisted candidates placed vertically based on the group ranking of importance from meeting 1 and horizontally in terms of the feasibility of monitoring that morbidity.

The panel were then asked in a secret ballot to nominate morbidities for exclusion without further discussion and others for inclusion without further discussion. The panel discussed the remaining morbidities as a group.

For peer review only - http://bmjopen.bmj.com/site/about/guidelines.xhtml

After the panel meeting, a written summary of the discussion was circulated and an online poll conducted to obtain the group ranking of importance among the shortlisted candidate morbidities. The poll was conducted to elicit the views of panellists who weren't able to attend and to identify replacement morbidities if any of those selected were judged to be infeasible to monitor in routine practice. We used the online voting tool at www.crankit.io, which uses the same algorithm for robustly inferring group preferences from individual preference data that was used in the selection meetings and that is outlined in appendix 1.

Final review of selected morbidities by definition panel and chief investigators

The selected morbidities were then reviewed by the definition panel, which could veto inclusion of a selected morbidity if, after careful consideration and discussion with the chief investigators (VT, KB), it was deemed infeasible to define and monitor in routine practice.

RESULTS

The panel convened is given at appendix 2. Of the 15 panellists, 3 could not attend the first meeting and 6 could not attend the second. An overview of the results of the selection process is given in figure 2.

FIGURE 2 HERE

First panel meeting:

At the first meeting, 66 morbidity terms were suggested during the round of 2 minute contributions from each panellist (see appendix 3). In the discussion that followed, 7 terms were removed as being irrelevant to our study, related to impacts of post-surgical morbidity that would either be measured as part of the our empirical study or were too long-term in nature to be captured within our study, or redundant given other terms suggested.

Of the remaining 59, 7 were accepted as candidate morbidities as they were and a further 5 were simply relabelled. The remaining 47 terms were mapped onto 16 groups of 2 – 8 terms, with each group considered to relate to a sufficiently similar phenomenon for them to be a single candidate morbidity. Members of the panel confirmed that it wanted the term "ECMO / Mechanical Support" to feature on its own as well as being an indicator of a "Major Adverse Event". A new term ("liver injury") was added as a candidate at this stage with the agreement of the panel. This gave 29 candidate morbidities.

These candidate morbidities and the group ranking of importance among them in the first round of voting are shown in table 1. It is worth noting that, in this first round of voting, the group ranked "new global permanent neurological impairment" as the most important morbidity. After that there was a group of 22 candidate morbidities that could only be separated by applying tie-breaks, with the other six candidate morbidities (including "necrotising enterocolitis") ranked as less important.

TABLE 1 HERE

In the discussion that followed the first round of voting, the panel merged the two items describing neurological impairment and individual panellists expressed surprise at the low ranking of "necrotising

For peer review only - http://bmjopen.bmj.com/site/about/guidelines.xhtml

enterocolitis” and “vascular thrombosis”, leading to a discussion of these particular morbidities and their impact.

The results of the second round of voting are shown in table 2. While the panel’s view of the top three morbidities (including the merged item of “New permanent neurological impairment (global or focal)) was clear, there was a large group of 21 candidates that could only be separated by applying tie-breaks. This group included “Necrotising enterocolitis” and “Vascular thrombosis”. Given the lack of unambiguous group preference among these 21, the panel decided to request that the separate definition panel consider the 24 candidate morbidities given in bold in table 2, with the remainder discarded.

TABLE 2 HERE

Second panel meeting:

The initial assessment of the definition panel as to the feasibility of defining and monitoring each candidate morbidity still in contention after the first selection panel meeting is shown to the right of figure 2, and as presented to the panel in appendix 4.

Eleven candidate morbidities were considered straightforward to define and monitor in routine practice: “unplanned reoperation / reintervention”, “length of ICU stay”, “Major adverse event (eg cardiac arrest, ECMO, Serious Untoward Incident)”, “ECMO / mechanical support”, “necrotising enterocolitis”, “prolonged hospital length of stay”, “acute kidney injury”, “prolonged pleural effusion”, “vascular thrombosis”, “surgical bleeding” and “complete heart block”.

Six candidate morbidities were considered less straightforward: “new permanent neurological impairment (global or focal)”, “problems feeding”, “hospital acquired infection”, “poor communication between clinical team and family”, “complications during surgery”, “phrenic nerve injury”.

The remaining 7 candidate morbidities were deemed difficult to define and monitor in routine practice: “new impaired cognitive function more than a month after surgery”, “developmental delay”, “low cardiac output”, “mental health consequences”, “recurrent laryngeal nerve palsy”, “questionable clinical team decision & diagnosis”, and “level of support at home”.

The output from the causal mapping exercises given at Appendix 5 highlighted how “mental health consequences” could be a result of several other candidate morbidities, how the majority of candidate morbidities could result in prolonged stay in intensive care or hospital and how “low cardiac output” could become manifest in several of the other candidate morbidities.

Based on these assessments and the selection panel’s own previous assessment of the importance of these morbidities, it was agreed that the following candidate morbidities would be selected without further discussion: “New permanent neurological impairment (global or focal)”, “unplanned reoperation / reintervention”, “problems feeding”, “acute kidney injury” and “poor communication between clinical team and family”.

The following candidate morbidities were discarded at this point without further discussion: “mental health consequences”, “prolonged hospital stay”, “questionable clinical team decision and diagnosis” and “level of support at home”.

For peer review only - http://bmjopen.bmj.com/site/about/guidelines.xhtml

The remaining 15 candidate morbidities were then discussed, with the panel reminded of the need to select at most 5 from these 15. Group decisions were made to select “ECMO”, “major adverse event”, “necrotising enterocolitis”, “hospital acquired infection” and “recurrent laryngeal nerve palsy”. Note that the group accepted that “Major adverse event” should not include ECMO as this had been selected as a distinct morbidity.

Group decisions were made to discard at this stage “new impaired cognitive function more than one month after surgery”, “developmental delay”, “low cardiac output”, “prolonged pleural effusion”, “vascular thrombosis”, “surgical bleeding”, “complications during surgery”, “phrenic nerve injury” and “length of ICU stay”.

The online poll conducted after the second panel meeting identified “Recurrent laryngeal nerve palsy”, “Phrenic nerve injury”, “Complete heart block” and “prolonged pleural effusion” as potential substitute morbidities in the event of the definition panel vetoing any of the selected morbidities in routine practice.

Final rulings of definitions panel and chief investigators

Of the ten morbidities selected by the panel, 2 (“poor communication between clinical team and family” and “recurrent laryngeal nerve palsy”) were removed from the final list by the definitions panel following input from and consultation with the chief investigators (VT and KB) and other members of the project management team. In each case this was done on the grounds that the morbidity concerned would be too problematic to measure in routine practice. The morbidity “prolonged pleural effusion” was added to the final list of morbidities as a substitute. Feeding problems due to symptomatic recurrent laryngeal nerve palsy were then included under “problems feeding”. The definition panel and the project team decided to monitor the incidence of phrenic nerve injury and complete heart block by using routinely collected cardiac audit data. These changes were shared with the selection panel.

The final nine morbidities chosen for inclusion in the study, using the revised labels used in working up final definitions are given in table 3. A detailed description of the definition process and the final definitions used is available at [31].

TABLE 3 HERE

Alongside these nine, the study team committed to measuring poor communication between the clinical team and the family among the 800+ patients anticipated to enter the matched cohort phase of our study and to conducting secondary analyses to identify the impact of longer stays in intensive care above and beyond the impact of the selected morbidities.

DISCUSSION

The morbidities selected to be measured in approximately 3600 patients over 18 months starting October 2015 cover a range of phenomena associated with paediatric cardiac surgery including indicators of organ damage such as acute kidney injury and necrotising enterocolitis, acute events such as cardiac arrest (included as a “major adverse event”), the necessity for major interventions in the intra-operative period such as ECMO, and problems feeding that, while not necessarily a priority during the intra-operative period, are considered to have a considerable impact on children and families in the months that follow.

For peer review only - http://bmjopen.bmj.com/site/about/guidelines.xhtml

At each stage of selection, the morbidity that was ranked of greatest importance by the panel was neurological impairment. This came as little surprise to the study investigators and vindicates the inclusion in our overarching programme of research of a parallel evaluation of the “Brief Developmental Assessment”, which it is hoped will provide a tool that can be deployed by nursing staff to identify patients that would benefit from referral to specialist neurological or other developmental services. However, it is fair to say that inclusion on the panel of family representatives and clinicians from outside the tertiary surgical centres brought other issues such as problems feeding and poor communication between clinical teams and families to greater prominence than if the panel had consisted solely of tertiary clinicians or if the study investigators had chosen the morbidities themselves.

We found that opening up the process of choosing the metrics by which services should monitor their performance to include the perspectives of patients and family representatives, which is in line with policy initiatives in England (32), brought challenges. Throughout our work, there was a tension between choosing a “clean” set of “clinical” measures that most closely matched the understanding of “surgical morbidity” among the tertiary clinicians on the panel and the inclusion of arguably murkier phenomena considered hugely important by families and those working in secondary care.

In particular, those working in surgical centres were more concerned than family representatives and others with the attribution of morbidity to the surgical act, keen to include morbidities that may be related in part to surgical technique (laryngeal nerve palsy and phrenic nerve injury) and degree of success (low cardiac output) and anxious to avoid the attribution to surgical teams of morbidities that are currently considered to “come with the territory” of congenital heart disease and its surgical treatment. Family representatives and others highlighted the value of gathering information on the incidence and impact of key morbidities, even if they were not caused by surgery, not least as some of them may be reducible through interventions at other points in the care pathway.

Strengths and limitations

We consider that several features of our study design were key to drawing out and balancing these perspectives. The Nominal Group Technique, starting as it does with an opportunity for each panellist to speak without interruption and within an embedded democratic process, is specifically designed to minimise the influence of perceived power differentials and dominant personalities within a group. This was reinforced by the use of a secret ballot process to determine group preferences, allowing panellists to record their disagreement with the positions stated by others without that being openly declared. The voting tool used distinguishes between unambiguous group preferences and those that rely on tie-breaking. This acceptance and presentation of lack of consensus helped to focus discussion on where it would be most valuable to the task of selecting a group of morbidities and divert unnecessary debate focused on achieving a false consensus through attrition.

Also, our choice to separate the process of identifying which morbidities are most important from the process of assessing the feasibility of defining and monitoring morbidities prevented all parties from self-censoring and clinical panellists from unconsciously using or claiming privileged knowledge of measurement processes to strengthen the case for morbidities they wanted to include.

Firm and expert chairing was essential to maintaining this discipline. Having a chair conversant with the clinical area but also experienced in working with multi-stakeholder groups including patient and family representatives was also key. Although it meant that the Chair brought their own clinical perspective to the table, the panel benefitted from the Chair’s ability to discern between the wheat and chaff of clinical discussions and summarise for non-clinical participants. It is also questionable whether a Chair that was

For peer review only - http://bmjopen.bmj.com/site/about/guidelines.xhtml

not an accomplished surgeon and clinical researcher would have held the respect of all parties through the process.

Separating the processes of judging the importance and the feasibility of routinely monitoring morbidities did however risk some of the subtlety of discussions slipping through the gaps between two panels of people. Whilst the preparation of detailed summaries of panel meetings and the presence of the same facilitating team (CP and MU) at all meetings reduced this risk, we acknowledge that the defined morbidities that will be monitored do not correspond exactly in all cases to the phenomena deemed important by the selection panel.

Another limitation of the face-to-face approach adopted is that the panel was necessarily of limited size and we cannot claim the priorities and preferences expressed are representative of the respective professional groups and of families in general. For instance, the perspectives of families were represented by three individuals. That said, all three were aware of the concerns of the broader population of families through roles with a parent-led charity or from facilitating one of the focus groups that fed into the selection process.

In summary, we found that the inclusion of patient and family perspectives in identifying metrics for use in monitoring a highly specialised clinical service is a challenging but feasible exercise that can add valuable breadth to notions of quality and how to measure it.

CONTRIBUTORSHIP STATEMENT

CP helped design the study, organised and facilitated the panels, contributed to analysis, contributed to the first draft of the paper and approved the final draft. KB helped design the study as co-chief investigator, contributed to the drafting of the paper and approved the final draft. IM contributed to the causal mapping exercise and approved the final draft. HJ contributed to the causal mapping exercise and approved the final draft. JW helped design the study, contributed to the drafting of the paper and approved the final draft. LC reviewed literature for the selection panel and approved the final draft. AM chaired the definition panel that fed into the work and approved the final draft. TT chaired the selection panel, contributed to the drafting of the manuscript and approved the final draft. VT helped design the study as co-chief investigator, contributed to the panel and approved the final draft. MU helped design the study, facilitated the panels, conducted the causal mapping exercise, contributed to analysis, wrote the first draft of the paper and approved the final draft.

COMPETING INTERESTS

MU and CP are part of the team that developed the online voting tool CRANKIT used in this study.

FUNDING

This project was funded by the National Institute for Health Research
Health Services and Delivery Research Programme (project number 12/5005/06).

MU was in part supported by the National Institute for Health Research (NIHR) Collaboration for Leadership in Applied Health Research and Care (CLAHRC) North Thames at Bart's Health NHS Trust.

KB, JW and VT were supported by the National Institute for Health Research Biomedical Research Centre at Great Ormond Street Hospital for Children NHS Foundation Trust and University College London.

The views and opinions expressed are those of the authors and do not necessarily reflect those of the Health Services and Delivery Research Programme, NIHR, NHS or the Department of Health.

For peer review only - http://bmjopen.bmj.com/site/about/guidelines.xhtml

DATA SHARING AGREEMENT

There are no additional data available.

ACKNOWLEDGEMENTS

We would like to thank Rehana Ahmed, David Barron, Jane Cassidy, Kate English, Allan Goldman, Samantha Johnson, Ravi Kumar, Andrew Parry, Samana Schwank, Emma Simmonds, Rob Tulloh and Emma Twigg for their contribution to this work.

REFERENCES

1. Aylin P, Alves B, Best N, Cook A, Elliott P, Evans SJ, et al. Comparison of UK paediatric cardiac surgical performance by analysis of routinely collected data 1984–96: was Bristol an outlier? The Lancet. 2001 Jul;358(9277):181–7.

2. Crowe S, Brown KL, Pagel C, Muthialu N, Cunningham D, Gibbs J, et al. Development of a diagnosis- and procedure-based risk model for 30-day outcome after pediatric cardiac surgery. J Thorac Cardiovasc Surg. 2013 May;145(5):1270–8.

3. Jacobs JP, O'Brien SM, Pasquali SK, Jacobs ML, Lacour-Gayet FG, Tchervenkov CI, et al. Variation in outcomes for risk-stratified pediatric cardiac surgical operations: an analysis of the STS Congenital Heart Surgery Database. Ann Thorac Surg. 2012 Aug;94(2):564-571-572.

4. Jenkins KJ, Gauvreau K, Newburger JW, Spray TL, Moller JH, Iezzoni LI. Consensus-based method for risk adjustment for surgery for congenital heart disease. J Thorac Cardiovasc Surg. 2002 Jan;123(1):110–8.

5. Kang N, Cole T, Tsang V, Elliott M, de Leval M. Risk stratification in paediatric open-heart surgery. Eur J Cardio-Thorac Surg Off J Eur Assoc Cardio-Thorac Surg. 2004 Jul;26(1):3–11.

6. Kang N, Tsang VT, Gallivan S, Sherlaw-Johnson C, Cole TJ, Elliott MJ, et al. Quality assurance in congenital heart surgery. Eur J Cardio-Thorac Surg Off J Eur Assoc Cardio-Thorac Surg. 2006 May;29(5):693-697-698.

7. Lacour-Gayet F, Clarke D, Jacobs J, Gaynor W, Hamilton L, Jacobs M, et al. The Aristotle score for congenital heart surgery. Semin Thorac Cardiovasc Surg Pediatr Card Surg Annu. 2004;7(1):185–91.

8. O'Brien SM, Clarke DR, Jacobs JP, Jacobs ML, Lacour-Gayet FG, Pizarro C, et al. An empirically based tool for analyzing mortality associated with congenital heart surgery. J Thorac Cardiovasc Surg. 2009 Nov;138(5):1139–53.

9. Pagel C, Brown KL, Crowe S, Utley M, Cunningham D, Tsang V. A mortality risk model to adjust for case mix in UK paediatric cardiac surgery. Health Serv Deliv Res. 2013 May;1(1).

10. Pagel C, Utley M, Crowe S, Witter T, Anderson D, Samson R, et al. Real time monitoring of risk-adjusted paediatric cardiac surgery outcomes using variable life-adjusted display: implementation in three UK centres. Heart Br Card Soc. 2013 Apr 5;

11. Cunningham D, Franklin R, Bridgewater B, Deanfield JE. Investigation of mortality from Paediatric Cardiac Surgery in England 2009-12: 8 April 2013 [Internet]. 2013 [cited 2013 Aug 17]. Available from: http://www.ucl.ac.uk/nicor/paediatric_cardiac_surgery_report

12. Jacobs ML, Jacobs JP, Franklin RCG, Mavroudis C, Lacour-Gayet F, Tchervenkov CI, et al. Databases for assessing the outcomes of the treatment of patients with congenital and paediatric cardiac disease--the perspective of cardiac surgery. Cardiol Young. 2008 Dec;18 Suppl 2:101–15.

For peer review only - http://bmjopen.bmj.com/site/about/guidelines.xhtml

13. Jenkins KJ, Beekman Iii RH, Bergersen LJ, Everett AD, Forbes TJ, Franklin RCG, et al. Databases for assessing the outcomes of the treatment of patients with congenital and paediatric cardiac disease--the perspective of cardiology. Cardiol Young. 2008 Dec;18 Suppl 2:116–23.

14. Shahian DM, Edwards FH, Jacobs JP, Prager RL, Normand S-LT, Shewan CM, et al. Public Reporting of Cardiac Surgery Performance: Part 1--History, Rationale, Consequences. Ann Thorac Surg. 2011 Sep 1;92(3_Supplement):S2-11.

15. Boseley S, editor health. Leeds General Infirmary to resume children's heart surgery. The Guardian [Internet]. 2013 Apr 8 [cited 2014 Mar 20]; Available from: http://www.theguardian.com/society/2013/apr/08/leeds-general-childrens-heart-surgery

16. Bower H. London hospital orders inquiry into paediatric heart surgery deaths. BMJ. 1999 Aug 7;319(7206):335–335.

17. Dyer C. Consultation over children's heart surgery was unfair to Royal Brompton, judge rules. BMJ. 2011 Nov 8;343(nov08 2):d7275–d7275.

18. Mayor S. Oxford hospital suspends paediatric cardiac surgery after four deaths. BMJ. 2010 Mar 4;340(mar04 2):c1303–c1303.

19. McGauran A. Fewer hospitals in England should perform children's heart surgery. BMJ. 2011 Feb 18;342(feb18 1):d1129–d1129.

20. Murphy JF. The Bristol Royal Infirmary Inquiry 18th July 2001. Ir Med J. 2001 Sep;94(8):228.

21. Brown KL, Crowe S, Franklin R, McLean A, Cunningham D, Barron D, et al. Trends in 30-day mortality rate and case mix for paediatric cardiac surgery in the UK between 2000 and 2010. Open Heart. 2015;2(1):e000157.

22. Belliveau D, Burton HJ, O'Blenes SB, Warren AE, Hancock Friesen CL. Real-time complication monitoring in pediatric cardiac surgery. Ann Thorac Surg. 2012 Nov;94(5):1596–602.

23. Cooke M, Thackray S. Differences between community professional and patient perceptions of chronic obstructive pulmonary disease treatment outcomes: a qualitative study. J Clin Nurs. 2012 Jun;21(11–12):1524–33.

24. NIHR Evaluation, Trials and Studies | 12/5005/06 [Internet]. [cited 2014 Apr 3]. Available from: http://www.nets.nihr.ac.uk/projects/hsdr/12500506

25. Gallagher M, Hares T, Spencer J, Bradshaw C, Webb I. The Nominal Group Technique: A Research Tool for General Practice? Fam Pract. 1993 Mar 1;10(1):76–81.

26. Jones J, Hunter D. Consensus methods for medical and health services research. BMJ. 1995 Aug 5;311(7001):376–80.

27. Mullen PM. Delphi: myths and reality. J Health Organ Manag. 2003;17(1):37–52.

28. Keeney S, Hasson F, McKenna HP. A critical review of the Delphi technique as a research methodology for nursing. Int J Nurs Stud. 2001 Apr;38(2):195–200.

29. Utley M, Gallivan S, Mills M, Mason M, Hargraves C. A consensus process for identifying a prioritised list of study questions. Health Care Manag Sci. 2007 Feb;10(1):105–10.

30. HS&DR - 12/5005/06: Selection, definition and evaluation of important early morbidities associated with paediatric cardiac surgery [Internet]. [cited 2014 Nov 4]. Available from: http://www.nets.nihr.ac.uk/projects/hsdr/12500506

For peer review only

31 Brown KL, Pagel C, Brimmell R, Bull K, Davis P, Franklin RC, Hoskote A, Khan N, Rodrigues W, Thorne S, Smith L, Chigaru L, Utley M, Wray J, Tsang V, Mclean A, Definition of important early morbidities related to paediatric cardiac surgery, Cardiology in the Young. 2016 Sep 29:1-10.

32. NHS England » Patient Centred Outcome Measures [Internet]. [cited 2016 Jan 3]. Available from: https://www.england.nhs.uk/ourwork/pe/pcoms/

For peer review only

FIGURES AND TABLES

Figure 1: the role of the selection panel within our wider study to identify and measure the incidence and impact of important morbidities following paediatric cardiac surgery

Figure 2: overview of results of selection process

For peer review only

Table 1- results of the first ranking exercise

Morbidities	Rank before tie-breaks	Rank after tie-breaks
New global permanent neurological impairment	1	1
New impaired cognitive function more than a month after surgery	2	2
Unplanned reoperation / reintervention	2	3
Developmental delay	2	4
Major adverse event	2	5
Problems feeding (graded)	2	6
Mental health consequences	2	7
Length of ICU stay	2	8
New focal permanent neurological impairment	2	9
Low cardiac output (categorised)	2	10
Poor communication between clinical team and family	2	11
ECMO / mechanical support	2	12
Acute kidney injury (graded)	2	13
Prolonged pleural effusion	2	14
Complications during surgery	2	15
Hospital acquired infection (graded/categorised)	2	15
Prolonged hospital length of stay	2	17
Complete heart block (categorised)	2	18
Surgical bleeding (graded)	2	19
Questionable clinical team decision & diagnosis	2	20
Level of support from hospital available at home	2	21
Recurrent laryngeal nerve palsy	2	21
Phrenic nerve injury	2	23
Necrotising enterocolitis	24	24
Vascular thrombosis (graded)	25	25
JET	26	26
Elevated pulmonary vascular resistance	27	27
Liver injury (graded)	27	27
Sensory neural deafness	27	27

For peer review only - http://bmjopen.bmj.com/site/about/guidelines.xhtml

Table 2 – Results of the second round of ranking. The morbidities passed to the definition panel for consideration are shown in **bold**.

Morbidities	Rank before tie-breaks	Rank after tie-breaks
**New permanent neurological impairment (global or focal)**	1	1
**New impaired cognitive function more than a month after surgery**	2	2
**Unplanned reoperation / reintervention (categorisation)**	3	3
**Length of ICU stay**	4	4
**Major adverse event**	4	5
**Problems feeding (graded)**	4	6
**Developmental delay**	4	7
**ECMO / mechanical support**	4	8
**Low cardiac output (categorised)**	4	8
**Mental health consequences**	4	10
**Necrotising enterocolitis**	4	11
**Hospital acquired infection (graded/categorised)**	4	12
**Prolonged hospital length of stay**	4	13
**Acute kidney injury (graded)**	4	14
**Prolonged pleural effusion**	4	15
**Poor communication between clinical team and family**	4	16
**Recurrent laryngeal nerve palsy**	4	17
**Vascular thrombosis (graded)**	4	18
**Surgical bleeding (graded)**	4	19
**Complications during surgery**	4	20
**Complete heart block (categorised)**	4	21
**Questionable clinical team decision & diagnosis**	4	22
**Phrenic nerve injury**	4	23
**Level of support at home**	4	24
JET	25	25
Sensory neural deafness	26	26
Elevated pulmonary vascular resistance	27	27
Liver injury (graded)	27	27

For peer review only - http://bmjopen.bmj.com/site/about/guidelines.xhtml

Table 3 – The final list of 9 selected morbidities

Morbidities
Acute neurological event
Unplanned reoperation / re-intervention
Problems feeding (excluding NEC)
Need for renal replacement therapy (excluding ECMO)
Major adverse event
ECLS / ECMO
Necrotising enterocolitis (NEC)
Hospital acquired infection (graded/categorised)
Prolonged pleural effusion / chylothorax

For peer review only

For peer review only - http://bmjopen.bmj.com/site/about/guidelines.xhtml

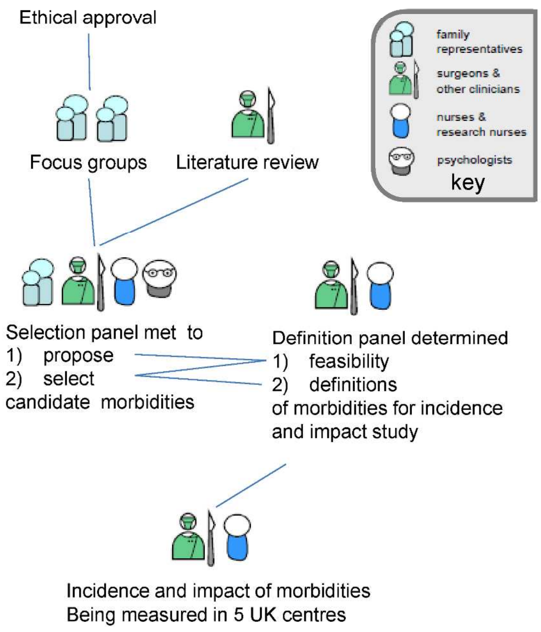

the role of the selection panel within our wider study to identify and measure the incidence and impact of important morbidities following paediatric cardiac surgery
Figure 1
254x190mm (300 x 300 DPI)

For peer review only

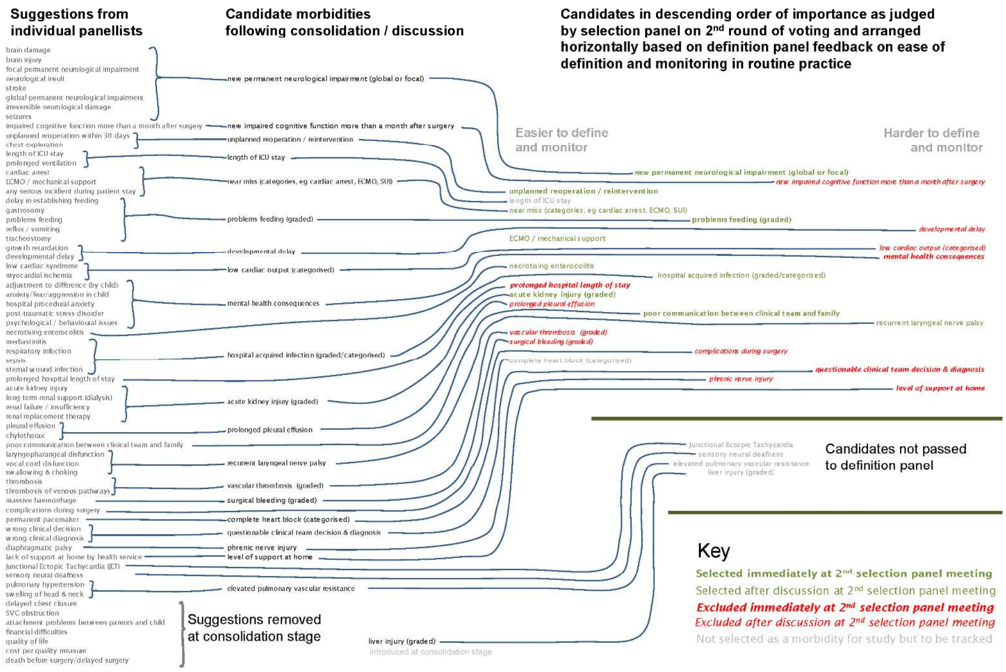

overview of results of selection process
Figure 2
254x190mm (300 x 300 DPI)

Appendix 1

Voting process used in selection

The voting process used as part of the nominal group technique deployed was based on the algorithm presented in *Utley M, Gallivan S, Mills M, Mason M, Hargraves C. A consensus process for identifying a prioritised list of study questions. Health Care Manag Sci. 2007 Feb;10(1):105–10*. A short summary is presented here.

In a secret ballot, each panellist ranked the candidate options in order of descending importance. They were permitted to use tied ranks and were not obliged to rank every option. For each and every possible pair of options (candidate morbidities A and B say) we determined whether at least as many participants preferred option A to option B as preferred option B to option A. We then used the analysis summarised in Box 1 to identify group preferences among the set of candidate morbidities.

In the first panel meeting, the rankings supplied by individual panellists were entered into an Excel spreadsheet and a VBA Macro written by MU and CP was used to conduct the analysis. For the online poll conducted after the second panel meeting, we used the web-based tool available at www.crankit.io.

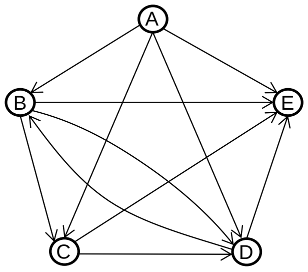

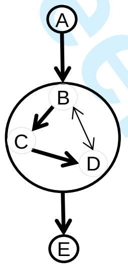

Rank before tie-breaks	Option	Rank after tie-breaks
1	A	1
2	B	2
2	C	3
2	D	4
5	E	5

Step 1

Draw an arrow from option A to B if at least as many participants ranked A higher than B as ranked B higher than A. Repeat process in each direction for all pairs of options.

In this example there is an arrow in both directions between B and D, indicating that exactly as many participants preferred B to D as preferred D to B…

Step 2

Identify the hierarchy of subsets of options. Within a subset there is no clear group ranking of options.

…there is a majority preference for A above every other option and E is least favoured. The cycle of arrows between B, C and D indicates that there is no unambiguous ranking of these 3...

Step 3

Try to rank options within subsets by first counting the arrows leaving each option within that subset and then, if there are ties, counting just those arrows between tied options.

… there are two arrows leaving B and just 1 leaving each of C and D, so the first tie-break places B above C and D. Then, considering just C and D, the second tie break places C above D.

Box 1; explanation of the robust group ranking process used in the nominal group approach employed by the selection panel.

For peer review only

Appendix 2

Panel composition

Name	Organisation	Role on panel
Ms Rehana Ahmed*	Independent	Family representative
Mr David Barron	Birmingham Children's Hospital	Heart surgeon
Dr Jane Cassidy†	Birmingham Children's Hospital	Intensive care doctor
Dr Kate English†	Leeds General Infirmary	Cardiologist
Dr Allan Goldman	Great Ormond Street Hospital	Intensive care doctor
Dr Helen Jepps†	Bradford Royal Infirmary	Paediatrician
Ms Samantha Johnson	Children's Heart Federation	Family representative
Dr Ravi Kumar*	Royal Berkshire Hospital	Paediatrician
Ms Isobel Macleod	Royal Hospital for Sick Children, Glasgow	Intensive care nurse
Mr Andrew Parry*	University Hospitals Bristol	Heart surgeon
Dr Samana Schwank	Children's Heart Federation	Family representative
Ms Emma Simmonds†	Evelina London Children's Hospital	Clinical nurse specialist
Professor Tom Treasure	Independent	Chairperson
Mr Victor Tsang	Great Ormond Street Hospital	Heart surgeon
Professor Rob Tulloh†	University Hospitals Bristol	Cardiologist
Dr Emma Twigg†	Great Ormond Street Hospital	Clinical psychologist

Table A2 Members of the selection panel. * indicates members who could not attend the 1st panel meeting, † those who could not attend the 2nd meeting. Members are listed in alphabetical order.

For peer review only

For peer review only - http://bmjopen.bmj.com/site/about/guidelines.xhtml

Appendix 3: The initial 66 terms under consideration in selection panel 1.

anxiety/fear/aggression	prolonged ventilation
problems feeding	complications during surgery
reflux/vomiting	developmental delay
pleural effusion	financial difficulties
swallowing & choking	lack of support at home
global permanent neurological impairment	laryngopharyngeal dysfunction
Mediastinitis	vocal cord dysfunction
myocardial ischemia	delay in establishing feeding
focal permanent neurological impairment	brain damage
sensory neural deafness	low cardiac output
brain injury	Sepsis
cardiac arrest	gastrosomy
renal failure/insufficiency	massive haemorrhage
length of ICU stay	thrombosis
post-traumatic stress disorder	impaired cognitive function more than a month after surgery
ECMO / mechanical support	quality of life
communication	prolonged hospital length of stay
hospital procedural anxiety	permanent pacemaker
adjustment to difference	long term renal support
Attachment	any serious incident during patient stay
neurological insult	cost per quality measure
Chylothorax	seizures
acute kidney injury	stroke
necrotising enterocolitis	sternal wound infection
junctional ectopic tachycardia (JET)	psychological/behavioural issues
pulmonary hypertension	growth retardation
SVC obstruction	tracheostomy
swelling of head & neck	diaphragmatic palsy
chest exploration	delayed chest closure
thrombosis of venous pathways	respiratory infection
irreversible neurological damage	wrong clinical decision
renal replacement therapy	wrong clinical diagnosis
unplanned reoperation within 30 days	death before surgery/delayed surgery

For peer review only - http://bmjopen.bmj.com/site/about/guidelines.xhtml

Appendix 4 - The summary of the feasibility of defining and measuring in routine practice (as judged by the definition panel) vs importance shown to the selection panel at their second meeting.

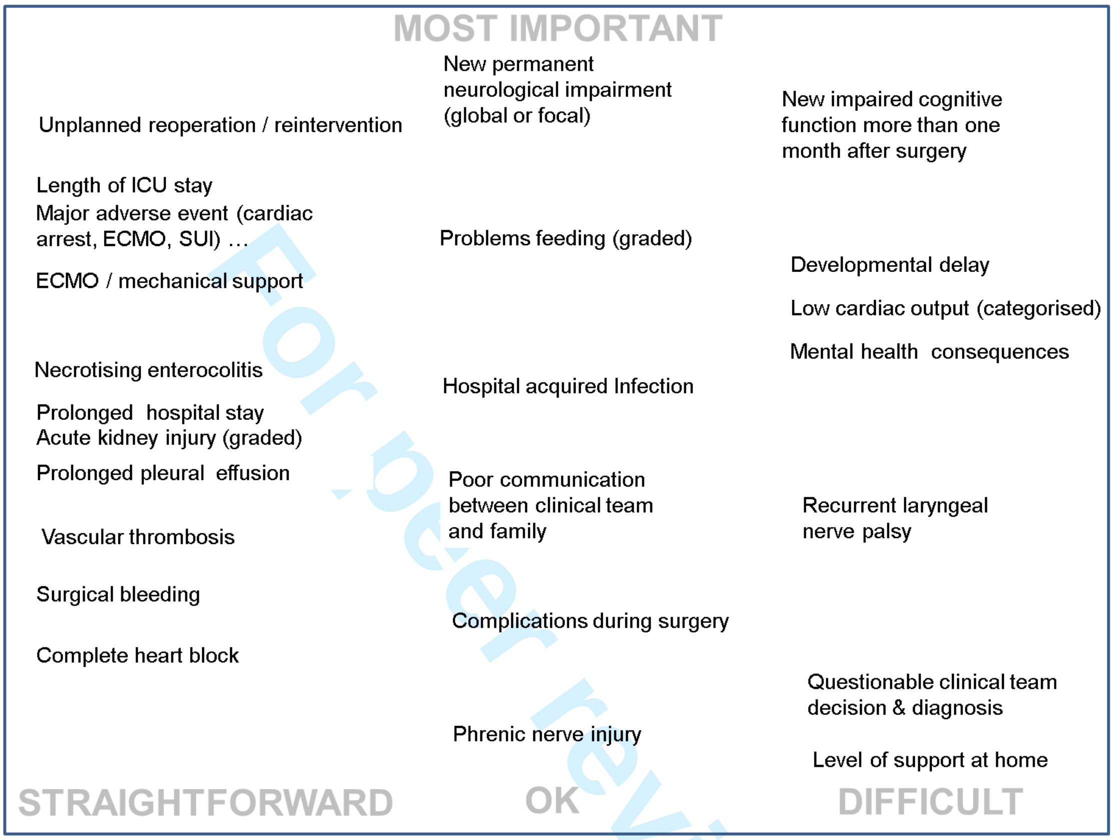

For peer review only

Appendix 5

Graphics shown to the selection panel at the second meeting on the relationships between candidate morbidities (figures A1-A3).

Figure A1

One view on relationship of other candidate morbidities to mental health consequences

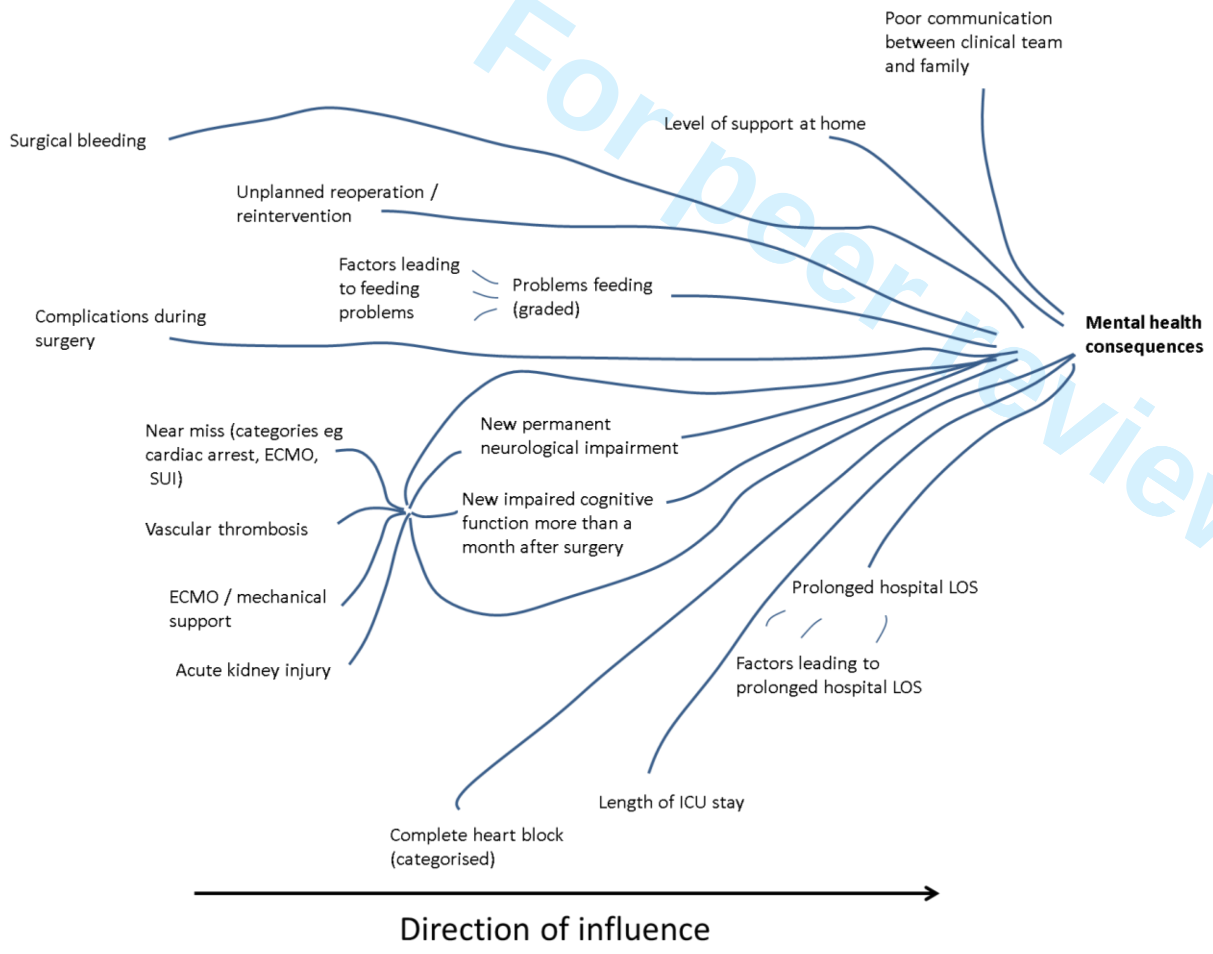

Figure A2

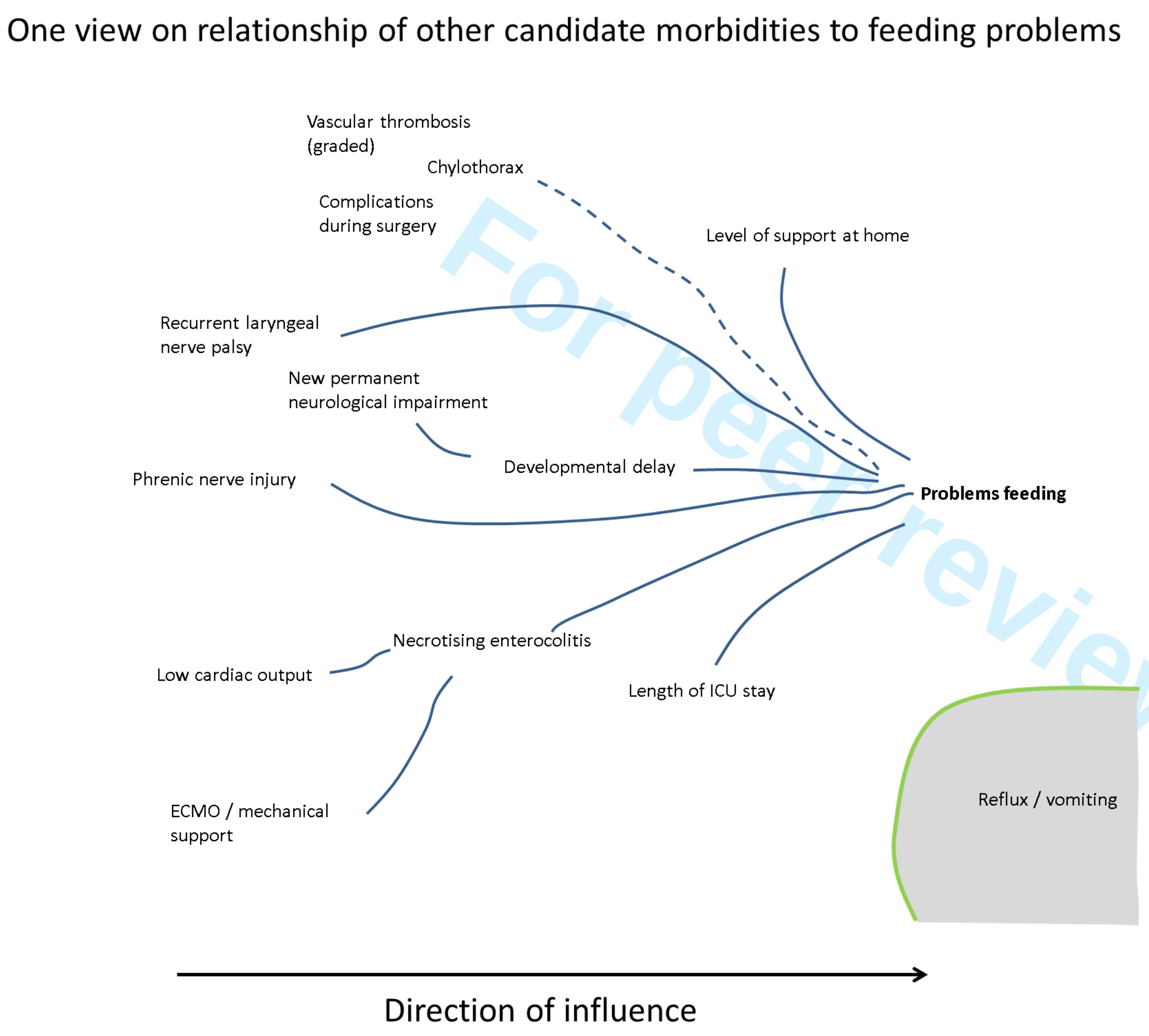
One view on relationship of other candidate morbidities to feeding problems
Vascular thrombosis (graded)
Chylothorax
Complications during surgery
Level of support at home
Recurrent laryngeal nerve palsy
New permanent neurological impairment
Developmental delay
Phrenic nerve injury
Problems feeding
Necrotising enterocolitis
Low cardiac output
Length of ICU stay
Reflux / vomiting
ECMO / mechanical support
Direction of influence

Figure A3

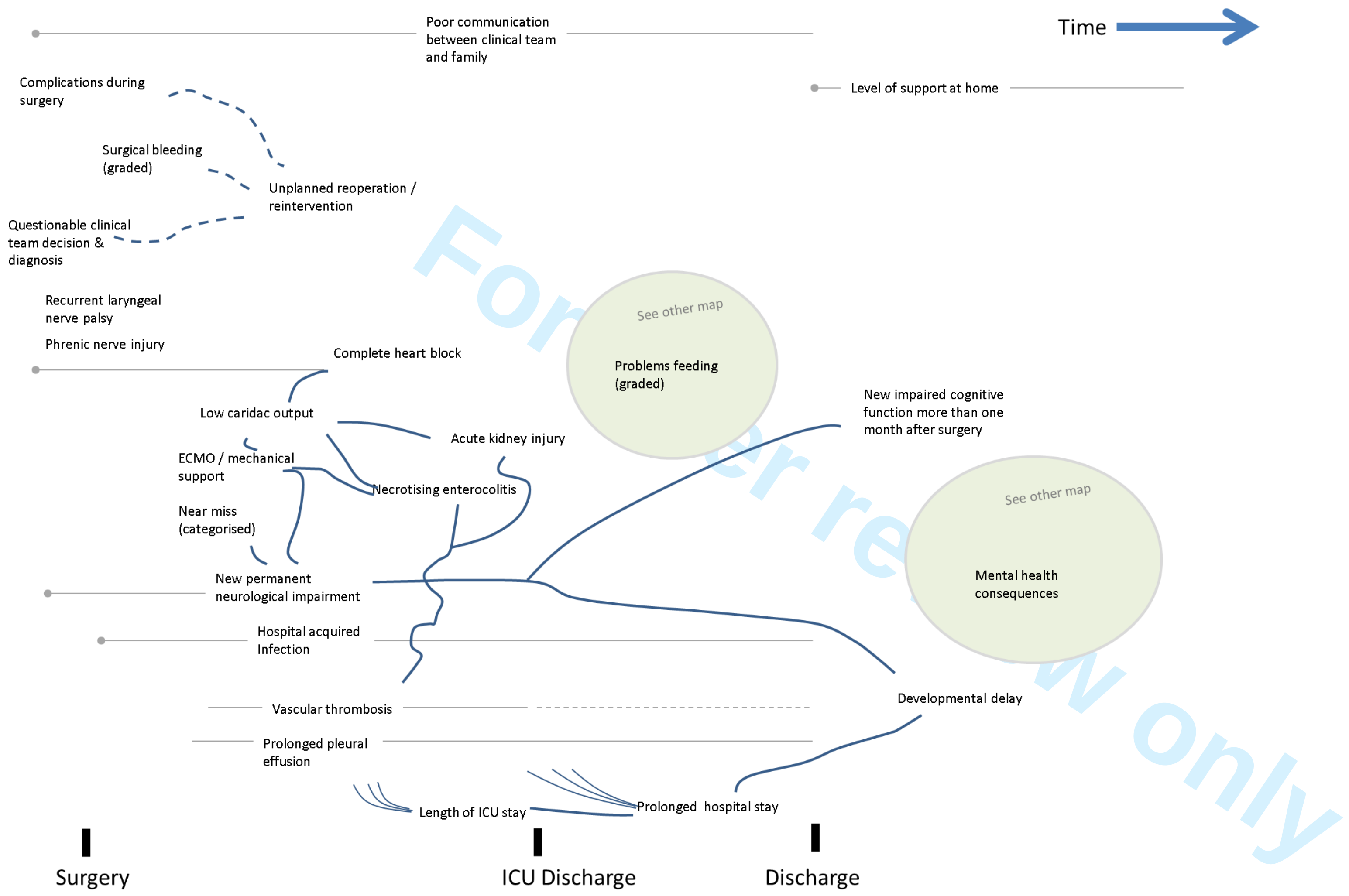

One view of whole picture (relationships of candidate morbidities to each other)

BMJ Open

Selection by a panel of clinicians and family representatives of important early morbidities associated with paediatric cardiac surgery suitable for routine monitoring using the nominal group technique and a robust voting process.

Journal:	*BMJ Open*
Manuscript ID	bmjopen-2016-014743.R2
Article Type:	Research
Date Submitted by the Author:	27-Feb-2017
Complete List of Authors:	Pagel, Christina; UCL, Clinical Operational Research Unit<br>Brown, Katherine; Great Ormond Street Hospital for Children, Cardiorespiratory Division<br>Macleod, Isobel; Royal Hospital for Children<br>Jepps, Helen; Bradford Teaching Hospitals NHS Foundation Trust<br>Wray, Jo; Great Ormond Street Hospital for Children, Cardiorespiratory Division<br>Chigaru, Linda; Great Ormond Street Hospital for Children, Cardiorespiratory Division<br>Mclean, Andrew; Royal Hospital for Children<br>Treasure, Tom; UCL, CORU Mathematics<br>Tsang, Victor; Great Ormond Street Hospital for Children, Cardiorespiratory Division<br>Utley, Martin; University College London, Clinical Operational Research Unit, Department of Mathematics
**Primary Subject Heading**:	Surgery
Secondary Subject Heading:	Patient-centred medicine, Research methods, Health services research
Keywords:	Paediatric cardiac surgery < PAEDIATRIC SURGERY, AUDIT, Quality in health care < HEALTH SERVICES ADMINISTRATION & MANAGEMENT

SCHOLARONE™ Manuscripts

Selection by a panel of clinicians and family representatives of important early morbidities associated with paediatric cardiac surgery suitable for routine monitoring using the nominal group technique and a robust voting process.

Authors: Christina Pagel, Katherine L Brown, Isobel McLeod, Helen Jepps, Jo Wray, Linda Chigaru, Andrew McLean, Tom Treasure, Victor Tsang, Martin Utley

Email address for corresponding author: m.utley@ucl.ac.uk

Abstract

Objective: With survival following paediatric cardiac surgery improving, the attention of quality assurance and improvement initiatives is shifting to long-term outcomes and early surgical morbidities. We wanted to involve family representatives and a range of clinicians in selecting the morbidities to be measured in a major UK study.

Setting: Paediatric cardiac surgery services in the UK.

Participants: We convened a panel comprising family representatives, paediatricians from referring centres and surgeons and other clinicians from surgical centres.

Primary and secondary outcome measures: Using the Nominal Group Technique augmented by a robust voting process to identify group preferences, suggestions for candidate morbidities were elicited, discussed, ranked and then shortlisted. The shortlist was passed to a clinical group that provided a view on the feasibility of monitoring each shortlisted morbidity in routine practice. The panel then met again to select a prioritised list of morbidities for further study, with the list finalised by the clinical group and chief investigators.

Results: At the first panel meeting, 66 initial suggestions were made, with this reduced to a shortlist of 24 after two rounds of discussion, consolidation and voting. At the second meeting, this shortlist was reduced to 10 candidate morbidities. Two were dropped on grounds of feasibility and replaced by another the panel considered important. The final list of 9 morbidities included indicators of organ damage, acute events and feeding problems. Family representatives and clinicians from outside tertiary centres brought some issues to greater prominence than if the panel had consisted solely of tertiary clinicians or study investigators.

Conclusion: The inclusion of patient and family perspectives in identifying metrics for use in monitoring a specialised clinical service is challenging but feasible and can broaden notions of quality and how to measure it.

Word counts: Abstract: 287 Main text**:** 3884.

For peer review only

Strengths and limitations

The Nominal Group Technique, augmented by a robust secret voting process, allowed us to incorporate the perspectives of family representatives and clinicians from different professional groups in selecting early surgical morbidities they felt important to monitor in routine practice.

The robust voting process used identified group preferences from the preferences expressed by individual panellists and identified where there was a lack of consensus, guiding further discussion.

One limitation of the approach adopted was that it reflied on firm and expert chairing.

There was some unresolved tension between selecting morbidities clearly attributable to the surgical act and morbidities that are important to families but can be considered to “come with the territory” of congenital heart disease and its management.

Relying on a face-to-face approach necessarily limited the size of the panel and we cannot claim the priorities and preferences expressed are representative of the respective professional groups and of families in general.

Introduction

Early mortality following paediatric cardiac surgery, defined as death within 30 days of surgery or death prior to discharge home, has been the focus of many research studies (1–10), audit initiatives (6,11–14) and, particularly in the UK, of public scrutiny (1,15–20) over recent decades. However, with early mortality having fallen to 2-3% (21), attention has shifted to longer term outcomes and to broadening the assessment of early outcomes to include early morbidities.

Reporting of early morbidities associated with paediatric cardiac surgery has been driven largely by the data available within the databases of professional societies (12) or those available within datasets curated by individual clinical teams (22). Quality assurance initiatives rooted in such datasets benefit greatly from considerable effort, often over many years, to agree definitions of the outcomes collected and design data collection processes. However, it is inevitable that the datasets agreed upon, constructed and curated by clinicians (as individuals or via professional societies) focus largely on outcomes considered important from the perspective of that clinician or professional group. Research in other specialties has shown that patients and carers can have quite different perceptions to clinicians on what outcomes are important to monitor as part of service evaluation (23).

We report here on a process used to select early morbidities as part of a study to identify and then measure the incidence and impact of important early morbidities among paediatric cardiac surgery patients (NIHR HS&DR 12/5005/06 (24)). A key aim of our work was to incorporate a broad set of perspectives, including those from family representatives and professionals from different sectors on what early morbidities were important to monitor in routine practice.

Methods

Overview

Figure 1 gives an overview of the role of our selection process within the wider study. A panel of clinicians and patient representatives met twice to shortlist and then to select early morbidities, the incidence and

For peer review only - http://bmjopen.bmj.com/site/about/guidelines.xhtml

impact of which they considered important to measure. As shown in Figure 1, our selection process was linked to a parallel process of defining potential morbidities. The shortlist of morbidities produced after the first meeting of the selection panel was considered by a separate group composed entirely of clinicians. This definition panel provided a view on the feasibility of defining, measuring and routinely monitoring each shortlisted morbidity, which informed the second selection meeting.

Figure 1 HERE

Composition of the selection panel

In forming the panel, we aimed to include clinicians from surgical centres, referring hospitals and primary care as well as family representatives. We wanted a panel of enough people to provide a range of perspectives while being manageable.

The panel comprised 15 people: 3 family representatives, 3 paediatric cardiac surgeons, 2 paediatric intensive care doctors, 2 paediatric cardiologists, 2 paediatricians, a paediatric intensive care nurse, a clinical nurse specialist and a clinical psychologist with experience of working with children with congenital heart disease and their families. The panel was chaired by a cardiothoracic surgeon with extensive experience of chairing multi-disciplinary panels. Two of the three family representatives were nominated by the Children's Heart Foundation, a parent-led charity and umbrella organisation of CHD charities and voluntary organisations. The third had facilitated one of the focus groups that fed into the selection process. We tried, but did not manage, to recruit a GP to the panel.

With the permission of panel members, both selection panel meetings were recorded and professionally transcribed. Each selection panel meeting also had a pre-determined seating plan to ensure that people from similar specialities were not grouped together.

Selection panel meeting 1: shortlisting

The aim of the first meeting was to identify a shortlist of 15-20 candidate morbidities that would then be considered by the definitions group. Prior to the meeting, the panel was supplied with an extensive list of candidate morbidities identified through:

- an ongoing systematic review conducted as part of our wider programme of research;
- three facilitated focus groups held in different UK cities with parents recruited by the Children's Heart Federation;
- an online forum for patients and families hosted on the website of the Children's Heart Federation.

The focus groups and literature review will be the subject of other publications. The panel was also sent an abridged version of the study protocol, a description of the role of the selection panel and an agenda. Each panellist was asked to identify among or beyond this list of candidate morbidities, those they judged most important to monitor routinely according to a deliberately broad working definition of a surgical morbidity as:

> *Any health or emotional problem that arose as a result of the fact of surgery (whether directly caused by surgery/post-operative care or not).*

For peer review only - http://bmjopen.bmj.com/site/about/guidelines.xhtml

For the first meeting, panellists were requested not to censor their suggestions on grounds of the perceived difficulty of definition or measurement and it was stressed that another group would be making these judgements.

We used the nominal group technique (25,26) augmented by a robust voting process to determine group rankings of morbidities. The nominal group technique is designed to reduce the influence of perceived power differentials and of dominant personalities on group decision making whilst retaining the benefit of discussion absent from other systematic approaches to group decision making such as Delphi (27,28). We inferred group preferences from individual rankings using a method developed by Utley et al (29) that is briefly described in appendix 1.

Structure of the first panel meeting

Each panellist was given the opportunity to speak uninterrupted for two minutes on the morbidities they considered important. Each suggestion was entered onto a spreadsheet which was projected in the meeting room to ensure accurate transcription. The panel was then given the opportunity to add to this initial list if they thought something important had been missed.

The Chair led a process of identifying suggestions that fell outside the working definition above, duplication among suggestions and merging of closely related suggestions. There was then a secret ballot in which panellists were asked individually to rank the resulting list of candidate suggestions in order of descending importance. The voting process and the method for generating group preferences from individual ranking data are described further in appendix 1.

During a scheduled break, the group preferences were calculated from the individual rankings by two of the authors (CP, MU) who did not have a vote on which morbidities to measure. After the break, the group preferences were fed back to the panel. The Chair then led a second round of discussion, focussing on the group preferences and giving panellists the opportunity to argue for specific morbidities being given greater importance, and for the group to further consolidate the list of morbidities.

There was a second round of secret ranking followed by feedback of group preferences prior to a consensus being sought as to the prioritised shortlist of 15 to 20 morbidities to be passed to the separate definitions panel for an assessment on the feasibility of defining, measuring and monitoring each in routine practice.

Causal mapping

Following the first selection group meeting, it was decided that it might be useful for non-clinical members of the panel to have an accessible summary of any causal relationships among candidate morbidities so that, when choosing the best set of morbidities to monitor, any overlap or redundancy among candidates could be accounted for. To this end, a set of causal mapping exercises was conducted by one of the facilitative team (MU) separately with two of the panellists (IM and HJ). In each exercise, cards representing shortlisted morbidities were placed on a large sheet of paper and arranged left to right with lines drawn to indicate potential causal relationships. Photographs were taken of these causal maps which were then converted to diagrams.

Selection panel meeting 2: incorporating feasibility and overlap.

Prior to the second meeting, panellists were provided with a pack of materials containing:

For peer review only - http://bmjopen.bmj.com/site/about/guidelines.xhtml

- a summary of any estimates of incidence and impact of candidate morbidities from the systematic review
- the judgment of the definition panel on the feasibility of defining, measuring and monitoring routinely each candidate morbidity
- summary of potential long term impacts of each candidate morbidity from the definition panel
- a summary of the parent and family focus groups and the online forum
- the diagrams generated through the causal mapping exercise.
- minutes from the first meeting
- a statement of the purpose of the second meeting and its agenda

At the beginning of the second meeting the panel were given a brief reminder of the scope of the overall project, the remit of the selection panel and the timescales we were working to.

The panel were tasked with narrowing the list of shortlisted candidates to a selection of 6-10, the incidence and impact of which would be measured in five centres over 18 months (30). It was explained that the upper limit of 10 morbidities was due to the sample size required for measuring the impact of distinct morbidities. It was explained that we could measure just the incidence of other morbidities if possible from routine data.

Panellists were also alerted to the (then) recently launched NHS England consultation on the future of Children's Heart Services in England, which highlighted the possibility of future national audit of surgical morbidities.

Panellists were asked to consider the following:

- *Feasibility of measurement, including timescales*. Given our plans to measure the impact of morbidities, the project team stressed that selected morbidities needed to be identifiable in a timely manner.
- *Overlap and redundancy among selected morbidities*. The project team made the point that selecting morbidities that almost always occur with other selected morbidities would pose problems in terms of measuring their individual impact. It highlighted that length of stay measures are particularly problematic in this respect.
- *Incidence*. Selected morbidities needed to have an incidence of at least 1.5-2% for us to measure their impact over 18 months due to sample-size considerations.

A summary of the judgements of the definition panel was presented to the panel, consisting of an array showing the shortlisted candidates placed vertically based on the group ranking of importance from meeting 1 and horizontally in terms of the feasibility of monitoring that morbidity.

The panel were then asked in a secret ballot to nominate morbidities for exclusion without further discussion and others for inclusion without further discussion. The panel discussed the remaining morbidities as a group.

For peer review only - http://bmjopen.bmj.com/site/about/guidelines.xhtml

After the panel meeting, a written summary of the discussion was circulated and an online poll conducted to obtain the group ranking of importance among the shortlisted candidate morbidities. The poll was conducted to elicit the views of panellists who weren't able to attend and to identify replacement morbidities if any of those selected were judged to be infeasible to monitor in routine practice. We used the online voting tool at www.crankit.io, which uses the same algorithm for robustly inferring group preferences from individual preference data that was used in the selection meetings and that is outlined in appendix 1.

Final review of selected morbidities by definition panel and chief investigators

The selected morbidities were then reviewed by the definition panel, which could veto inclusion of a selected morbidity if, after careful consideration and discussion with the chief investigators (VT, KB), it was deemed infeasible to define and monitor in routine practice.

Governance

The study "Selection, definition and evaluation of important early morbidities associated with paediatric cardiac surgery" received a Favourable Opinion from the NRES London City Road & Hampstead Research Ethics Committee on 8 November 2013 (REC reference 13/LO/1442).

RESULTS

The panel convened is given at appendix 2. Of the 15 panellists, 3 could not attend the first meeting and 6 could not attend the second. An overview of the results of the selection process is given in figure 2.

FIGURE 2 HERE

First panel meeting:

At the first meeting, 66 morbidity terms were suggested during the round of 2 minute contributions from each panellist (see appendix 3). In the discussion that followed, 7 terms were removed as being irrelevant to our study, related to impacts of post-surgical morbidity that would either be measured as part of the our empirical study or were too long-term in nature to be captured within our study, or redundant given other terms suggested.

Of the remaining 59, 7 were accepted as candidate morbidities as they were and a further 5 were simply relabelled. The remaining 47 terms were mapped onto 16 groups of 2 – 8 terms, with each group considered to relate to a sufficiently similar phenomenon for them to be a single candidate morbidity. Members of the panel confirmed that it wanted the term "ECMO / Mechanical Support" to feature on its own as well as being an indicator of a "Major Adverse Event". A new term ("liver injury") was added as a candidate at this stage with the agreement of the panel. This gave 29 candidate morbidities.

These candidate morbidities and the group ranking of importance among them in the first round of voting are shown in table 1. It is worth noting that, in this first round of voting, the group ranked "new global permanent neurological impairment" as the most important morbidity. After that there was a group of 22 candidate morbidities that could only be separated by applying tie-breaks, with the other six candidate morbidities (including "necrotising enterocolitis") ranked as less important.

For peer review only - http://bmjopen.bmj.com/site/about/guidelines.xhtml

TABLE 1 HERE

In the discussion that followed the first round of voting, the panel merged the two items describing neurological impairment and individual panellists expressed surprise at the low ranking of “necrotising enterocolitis” and “vascular thrombosis”, leading to a discussion of these particular morbidities and their impact.

The results of the second round of voting are shown in table 2. While the panel’s view of the top three morbidities (including the merged item of “New permanent neurological impairment (global or focal)) was clear, there was a large group of 21 candidates that could only be separated by applying tie-breaks. This group included “Necrotising enterocolitis” and “Vascular thrombosis”. Given the lack of unambiguous group preference among these 21, the panel decided to request that the separate definition panel consider the 24 candidate morbidities given in bold in table 2, with the remainder discarded.

TABLE 2 HERE

Second panel meeting:

The initial assessment of the definition panel as to the feasibility of defining and monitoring each candidate morbidity still in contention after the first selection panel meeting is shown to the right of figure 2, and as presented to the panel in appendix 4.

Eleven candidate morbidities were considered straightforward to define and monitor in routine practice: “unplanned reoperation / reintervention”, “length of ICU stay”, “Major adverse event (eg cardiac arrest, ECMO, Serious Untoward Incident)”, “ECMO / mechanical support”, “necrotising enterocolitis”, “prolonged hospital length of stay”, “acute kidney injury”, “prolonged pleural effusion”, “vascular thrombosis”, “surgical bleeding” and “complete heart block”.

Six candidate morbidities were considered less straightforward: “new permanent neurological impairment (global or focal)”, “problems feeding”, “hospital acquired infection”, “poor communication between clinical team and family”, “complications during surgery”, “phrenic nerve injury”.

The remaining 7 candidate morbidities were deemed difficult to define and monitor in routine practice: “new impaired cognitive function more than a month after surgery”, “developmental delay”, “low cardiac output”, “mental health consequences”, “recurrent laryngeal nerve palsy”, “questionable clinical team decision & diagnosis”, and “level of support at home”.

The output from the causal mapping exercises given at Appendix 5 highlighted how “mental health consequences” could be a result of several other candidate morbidities, how the majority of candidate morbidities could result in prolonged stay in intensive care or hospital and how “low cardiac output” could become manifest in several of the other candidate morbidities.

Based on these assessments and the selection panel’s own previous assessment of the importance of these morbidities, it was agreed that the following candidate morbidities would be selected without further discussion: “New permanent neurological impairment (global or focal)”, “unplanned reoperation /

For peer review only - http://bmjopen.bmj.com/site/about/guidelines.xhtml

reintervention", "problems feeding", "acute kidney injury" and "poor communication between clinical team and family".

The following candidate morbidities were discarded at this point without further discussion: "mental health consequences", "prolonged hospital stay", "questionable clinical team decision and diagnosis" and "level of support at home".

The remaining 15 candidate morbidities were then discussed, with the panel reminded of the need to select at most 5 from these 15. Group decisions were made to select "ECMO", "major adverse event", "necrotising enterocolitis", "hospital acquired infection" and "recurrent laryngeal nerve palsy". Note that the group accepted that "Major adverse event" should not include ECMO as this had been selected as a distinct morbidity.

Group decisions were made to discard at this stage "new impaired cognitive function more than one month after surgery", "developmental delay", "low cardiac output", "prolonged pleural effusion", "vascular thrombosis", "surgical bleeding", "complications during surgery", "phrenic nerve injury" and "length of ICU stay".

The online poll conducted after the second panel meeting identified "Recurrent laryngeal nerve palsy", "Phrenic nerve injury", "Complete heart block" and "prolonged pleural effusion" as potential substitute morbidities in the event of the definition panel vetoing any of the selected morbidities in routine practice.

Final rulings of definitions panel and chief investigators

Of the ten morbidities selected by the panel, 2 ("poor communication between clinical team and family" and "recurrent laryngeal nerve palsy") were removed from the final list by the definitions panel following input from and consultation with the chief investigators (VT and KB) and other members of the project management team. In each case this was done on the grounds that the morbidity concerned would be too problematic to measure in routine practice. The morbidity "prolonged pleural effusion" was added to the final list of morbidities as a substitute. Feeding problems due to symptomatic recurrent laryngeal nerve palsy were then included under "problems feeding". The definition panel and the project team decided to monitor the incidence of phrenic nerve injury and complete heart block by using routinely collected cardiac audit data. These changes were shared with the selection panel.

The final nine morbidities chosen for inclusion in the study, using the revised labels used in working up final definitions are given in table 3. A detailed description of the definition process and the final definitions used is available at [31].

TABLE 3 HERE

Alongside these nine, the study team committed to measuring poor communication between the clinical team and the family among the 800+ patients anticipated to enter the matched cohort phase of our study and to conducting secondary analyses to identify the impact of longer stays in intensive care above and beyond the impact of the selected morbidities.

DISCUSSION

The morbidities selected to be measured in approximately 3600 patients over 18 months starting October 2015 cover a range of phenomena associated with paediatric cardiac surgery including indicators of organ

For peer review only - http://bmjopen.bmj.com/site/about/guidelines.xhtml

damage such as acute kidney injury and necrotising enterocolitis, acute events such as cardiac arrest (included as a “major adverse event”), the necessity for major interventions in the intra-operative period such as ECMO, and problems feeding that, while not necessarily a priority during the intra-operative period, are considered to have a considerable impact on children and families in the months that follow.

At each stage of selection, the morbidity that was ranked of greatest importance by the panel was neurological impairment. This came as little surprise to the study investigators and vindicates the inclusion in our overarching programme of research of a parallel evaluation of the “Brief Developmental Assessment”, which it is hoped will provide a tool that can be deployed by nursing staff to identify patients that would benefit from referral to specialist neurological or other developmental services. However, it is fair to say that inclusion on the panel of family representatives and clinicians from outside the tertiary surgical centres brought other issues such as problems feeding and poor communication between clinical teams and families to greater prominence than if the panel had consisted solely of tertiary clinicians or if the study investigators had chosen the morbidities themselves.

We found that opening up the process of choosing the metrics by which services should monitor their performance to include the perspectives of patients and family representatives, which is in line with policy initiatives in England (32), brought challenges. Throughout our work, there was a tension between choosing a “clean” set of “clinical” measures that most closely matched the understanding of “surgical morbidity” among the tertiary clinicians on the panel and the inclusion of arguably murkier phenomena considered hugely important by families and those working in secondary care.

In particular, those working in surgical centres were more concerned than family representatives and others with the attribution of morbidity to the surgical act, keen to include morbidities that may be related in part to surgical technique (laryngeal nerve palsy and phrenic nerve injury) and degree of success (low cardiac output) and anxious to avoid the attribution to surgical teams of morbidities that are currently considered to “come with the territory” of congenital heart disease and its surgical treatment. Family representatives and others highlighted the value of gathering information on the incidence and impact of key morbidities, even if they were not caused by surgery, not least as some of them may be reducible through interventions at other points in the care pathway.

Strengths and limitations

We consider that several features of our study design were key to drawing out and balancing these perspectives. The Nominal Group Technique, starting as it does with an opportunity for each panellist to speak without interruption and within an embedded democratic process, is specifically designed to minimise the influence of perceived power differentials and dominant personalities within a group. This was reinforced by the use of a secret ballot process to determine group preferences, allowing panellists to record their disagreement with the positions stated by others without that being openly declared. The voting tool used distinguishes between unambiguous group preferences and those that rely on tie-breaking. This acceptance and presentation of lack of consensus helped to focus discussion on where it would be most valuable to the task of selecting a group of morbidities and divert unnecessary debate focused on achieving a false consensus through attrition.

Also, our choice to separate the process of identifying which morbidities are most important from the process of assessing the feasibility of defining and monitoring morbidities prevented all parties from self-censoring and clinical panellists from unconsciously using or claiming privileged knowledge of measurement processes to strengthen the case for morbidities they wanted to include.

For peer review only - http://bmjopen.bmj.com/site/about/guidelines.xhtml

Firm and expert chairing was essential to maintaining this discipline. Having a chair conversant with the clinical area but also experienced in working with multi-stakeholder groups including patient and family representatives was also key. Although it meant that the Chair brought their own clinical perspective to the table, the panel benefitted from the Chair's ability to discern between the wheat and chaff of clinical discussions and summarise for non-clinical participants. It is also questionable whether a Chair that was not an accomplished surgeon and clinical researcher would have held the respect of all parties through the process.

Separating the processes of judging the importance and the feasibility of routinely monitoring morbidities did however risk some of the subtlety of discussions slipping through the gaps between two panels of people. Whilst the preparation of detailed summaries of panel meetings and the presence of the same facilitating team (CP and MU) at all meetings reduced this risk, we acknowledge that the defined morbidities that will be monitored do not correspond exactly in all cases to the phenomena deemed important by the selection panel.

Another limitation of the face-to-face approach adopted is that the panel was necessarily of limited size and we cannot claim the priorities and preferences expressed are representative of the respective professional groups and of families in general. For instance, the perspectives of families were represented by three individuals. That said, all three were aware of the concerns of the broader population of families through roles with a parent-led charity or from facilitating one of the focus groups that fed into the selection process.

In summary, we found that the inclusion of patient and family perspectives in identifying metrics for use in monitoring a highly specialised clinical service is a challenging but feasible exercise that can add valuable breadth to notions of quality and how to measure it.

CONTRIBUTORSHIP STATEMENT

CP helped design the study, organised and facilitated the panels, contributed to analysis, contributed to the first draft of the paper and approved the final draft. KB helped design the study as co-chief investigator, contributed to the drafting of the paper and approved the final draft. IM contributed to the causal mapping exercise and approved the final draft. HJ contributed to the causal mapping exercise and approved the final draft. JW helped design the study, contributed to the drafting of the paper and approved the final draft. LC reviewed literature for the selection panel and approved the final draft. AM chaired the definition panel that fed into the work and approved the final draft. TT chaired the selection panel, contributed to the drafting of the manuscript and approved the final draft. VT helped design the study as co-chief investigator, contributed to the panel and approved the final draft. MU helped design the study, facilitated the panels, conducted the causal mapping exercise, contributed to analysis, wrote the first draft of the paper and approved the final draft.

COMPETING INTERESTS

MU and CP are part of the team that developed the online voting tool CRANKIT used in this study.

FUNDING

This project was funded by the National Institute for Health Research
Health Services and Delivery Research Programme (project number 12/5005/06).

MU was in part supported by the National Institute for Health Research (NIHR) Collaboration for Leadership in Applied Health Research and Care (CLAHRC) North Thames at Bart's Health NHS Trust.

For peer review only - http://bmjopen.bmj.com/site/about/guidelines.xhtml

KB, JW and VT were supported by the National Institute for Health Research Biomedical Research Centre at Great Ormond Street Hospital for Children NHS Foundation Trust and University College London.

The views and opinions expressed are those of the authors and do not necessarily reflect those of the Health Services and Delivery Research Programme, NIHR, NHS or the Department of Health.

DATA SHARING AGREEMENT

There are no additional data available.

ACKNOWLEDGEMENTS

We would like to thank the panellists that contributed to this work, whose details can be found at http://www.gosh.nhs.uk/medical-information/clinical-specialties/cardiothoracic-surgery-information-parents-and-visitors/why-we-do-research/complications-after-heart-surgery-children.

REFERENCES

1. Aylin P, Alves B, Best N, Cook A, Elliott P, Evans SJ, et al. Comparison of UK paediatric cardiac surgical performance by analysis of routinely collected data 1984–96: was Bristol an outlier? The Lancet. 2001 Jul;358(9277):181–7.

2. Crowe S, Brown KL, Pagel C, Muthialu N, Cunningham D, Gibbs J, et al. Development of a diagnosis- and procedure-based risk model for 30-day outcome after pediatric cardiac surgery. J Thorac Cardiovasc Surg. 2013 May;145(5):1270–8.

3. Jacobs JP, O'Brien SM, Pasquali SK, Jacobs ML, Lacour-Gayet FG, Tchervenkov CI, et al. Variation in outcomes for risk-stratified pediatric cardiac surgical operations: an analysis of the STS Congenital Heart Surgery Database. Ann Thorac Surg. 2012 Aug;94(2):564-571-572.

4. Jenkins KJ, Gauvreau K, Newburger JW, Spray TL, Moller JH, Iezzoni LI. Consensus-based method for risk adjustment for surgery for congenital heart disease. J Thorac Cardiovasc Surg. 2002 Jan;123(1):110–8.

5. Kang N, Cole T, Tsang V, Elliott M, de Leval M. Risk stratification in paediatric open-heart surgery. Eur J Cardio-Thorac Surg Off J Eur Assoc Cardio-Thorac Surg. 2004 Jul;26(1):3–11.

6. Kang N, Tsang VT, Gallivan S, Sherlaw-Johnson C, Cole TJ, Elliott MJ, et al. Quality assurance in congenital heart surgery. Eur J Cardio-Thorac Surg Off J Eur Assoc Cardio-Thorac Surg. 2006 May;29(5):693-697-698.

7. Lacour-Gayet F, Clarke D, Jacobs J, Gaynor W, Hamilton L, Jacobs M, et al. The Aristotle score for congenital heart surgery. Semin Thorac Cardiovasc Surg Pediatr Card Surg Annu. 2004;7(1):185–91.

8. O'Brien SM, Clarke DR, Jacobs JP, Jacobs ML, Lacour-Gayet FG, Pizarro C, et al. An empirically based tool for analyzing mortality associated with congenital heart surgery. J Thorac Cardiovasc Surg. 2009 Nov;138(5):1139–53.

9. Pagel C, Brown KL, Crowe S, Utley M, Cunningham D, Tsang V. A mortality risk model to adjust for case mix in UK paediatric cardiac surgery. Health Serv Deliv Res. 2013 May;1(1).

10. Pagel C, Utley M, Crowe S, Witter T, Anderson D, Samson R, et al. Real time monitoring of risk-adjusted paediatric cardiac surgery outcomes using variable life-adjusted display: implementation in three UK centres. Heart Br Card Soc. 2013 Apr 5;

For peer review only - http://bmjopen.bmj.com/site/about/guidelines.xhtml

11. Cunningham D, Franklin R, Bridgewater B, Deanfield JE. Investigation of mortality from Paediatric Cardiac Surgery in England 2009-12: 8 April 2013 [Internet]. 2013 [cited 2013 Aug 17]. Available from: http://www.ucl.ac.uk/nicor/paediatric_cardiac_surgery_report

12. Jacobs ML, Jacobs JP, Franklin RCG, Mavroudis C, Lacour-Gayet F, Tchervenkov CI, et al. Databases for assessing the outcomes of the treatment of patients with congenital and paediatric cardiac disease--the perspective of cardiac surgery. Cardiol Young. 2008 Dec;18 Suppl 2:101–15.

13. Jenkins KJ, Beekman Iii RH, Bergersen LJ, Everett AD, Forbes TJ, Franklin RCG, et al. Databases for assessing the outcomes of the treatment of patients with congenital and paediatric cardiac disease--the perspective of cardiology. Cardiol Young. 2008 Dec;18 Suppl 2:116–23.

14. Shahian DM, Edwards FH, Jacobs JP, Prager RL, Normand S-LT, Shewan CM, et al. Public Reporting of Cardiac Surgery Performance: Part 1--History, Rationale, Consequences. Ann Thorac Surg. 2011 Sep 1;92(3_Supplement):S2-11.

15. Boseley S, editor health. Leeds General Infirmary to resume children's heart surgery. The Guardian [Internet]. 2013 Apr 8 [cited 2014 Mar 20]; Available from: http://www.theguardian.com/society/2013/apr/08/leeds-general-childrens-heart-surgery

16. Bower H. London hospital orders inquiry into paediatric heart surgery deaths. BMJ. 1999 Aug 7;319(7206):335–335.

17. Dyer C. Consultation over children's heart surgery was unfair to Royal Brompton, judge rules. BMJ. 2011 Nov 8;343(nov08 2):d7275–d7275.

18. Mayor S. Oxford hospital suspends paediatric cardiac surgery after four deaths. BMJ. 2010 Mar 4;340(mar04 2):c1303–c1303.

19. McGauran A. Fewer hospitals in England should perform children's heart surgery. BMJ. 2011 Feb 18;342(feb18 1):d1129–d1129.

20. Murphy JF. The Bristol Royal Infirmary Inquiry 18th July 2001. Ir Med J. 2001 Sep;94(8):228.

21. Brown KL, Crowe S, Franklin R, McLean A, Cunningham D, Barron D, et al. Trends in 30-day mortality rate and case mix for paediatric cardiac surgery in the UK between 2000 and 2010. Open Heart. 2015;2(1):e000157.

22. Belliveau D, Burton HJ, O'Blenes SB, Warren AE, Hancock Friesen CL. Real-time complication monitoring in pediatric cardiac surgery. Ann Thorac Surg. 2012 Nov;94(5):1596–602.

23. Cooke M, Thackray S. Differences between community professional and patient perceptions of chronic obstructive pulmonary disease treatment outcomes: a qualitative study. J Clin Nurs. 2012 Jun;21(11–12):1524–33.

24. NIHR Evaluation, Trials and Studies | 12/5005/06 [Internet]. [cited 2014 Apr 3]. Available from: http://www.nets.nihr.ac.uk/projects/hsdr/12500506

25. Gallagher M, Hares T, Spencer J, Bradshaw C, Webb I. The Nominal Group Technique: A Research Tool for General Practice? Fam Pract. 1993 Mar 1;10(1):76–81.

26. Jones J, Hunter D. Consensus methods for medical and health services research. BMJ. 1995 Aug 5;311(7001):376–80.

27. Mullen PM. Delphi: myths and reality. J Health Organ Manag. 2003;17(1):37–52.

28. Keeney S, Hasson F, McKenna HP. A critical review of the Delphi technique as a research methodology for nursing. Int J Nurs Stud. 2001 Apr;38(2):195–200.

For peer review only

29. Utley M, Gallivan S, Mills M, Mason M, Hargraves C. A consensus process for identifying a prioritised list of study questions. Health Care Manag Sci. 2007 Feb;10(1):105–10.

30. HS&DR - 12/5005/06: Selection, definition and evaluation of important early morbidities associated with paediatric cardiac surgery [Internet]. [cited 2014 Nov 4]. Available from: http://www.nets.nihr.ac.uk/projects/hsdr/12500506

31 Brown KL, Pagel C, Brimmell R, Bull K, Davis P, Franklin RC, Hoskote A, Khan N, Rodrigues W, Thorne S, Smith L, Chigaru L, Utley M, Wray J, Tsang V, Mclean A, Definition of important early morbidities related to paediatric cardiac surgery, Cardiology in the Young. 2016 Sep 29:1-10.

32. NHS England » Patient Centred Outcome Measures [Internet]. [cited 2016 Jan 3]. Available from: https://www.england.nhs.uk/ourwork/pe/pcoms/

For peer review only

For peer review only - http://bmjopen.bmj.com/site/about/guidelines.xhtml

FIGURES AND TABLES

Figure 1: the role of the selection panel within our wider study to identify and measure the incidence and impact of important morbidities following paediatric cardiac surgery

Figure 2: overview of results of selection process

For peer review only

For peer review only - http://bmjopen.bmj.com/site/about/guidelines.xhtml

Table 1- results of the first ranking exercise. Note that for some suggested morbidities, the panel indicated that a grading or categorisation scheme would be required if that morbidity were to be used. The definition of an appropriate grading or categorisation scheme was left to the separate definitions panel, with only those morbidities selected subject to the full definition process.

**Morbidities**	Rank before tie-breaks	Rank after tie-breaks
New global permanent neurological impairment	1	1
New impaired cognitive function more than a month after surgery	2	2
Unplanned reoperation / reintervention	2	3
Developmental delay	2	4
Major adverse event	2	5
Problems feeding (graded)	2	6
Mental health consequences	2	7
Length of ICU stay	2	8
New focal permanent neurological impairment	2	9
Low cardiac output (categorised)	2	10
Poor communication between clinical team and family	2	11
ECMO / mechanical support	2	12
Acute kidney injury (graded)	2	13
Prolonged pleural effusion	2	14
Complications during surgery	2	15
Hospital acquired infection (graded/categorised)	2	15
Prolonged hospital length of stay	2	17
Complete heart block (categorised)	2	18
Surgical bleeding (graded)	2	19
Questionable clinical team decision & diagnosis	2	20
Level of support from hospital available at home	2	21
Recurrent laryngeal nerve palsy	2	21
Phrenic nerve injury	2	23
Necrotising enterocolitis	24	24
Vascular thrombosis (graded)	25	25
JET	26	26
Elevated pulmonary vascular resistance	27	27
Liver injury (graded)	27	27
Sensory neural deafness	27	27

For peer review only - http://bmjopen.bmj.com/site/about/guidelines.xhtml

Table 2 – Results of the second round of ranking. The morbidities passed to the definition panel for consideration are shown in **bold**.

Morbidities	Rank before tie-breaks	Rank after tie-breaks
**New permanent neurological impairment (global or focal)**	1	1
**New impaired cognitive function more than a month after surgery**	2	2
**Unplanned reoperation / reintervention (categorisation)**	3	3
**Length of ICU stay**	4	4
**Major adverse event**	4	5
**Problems feeding (graded)**	4	6
**Developmental delay**	4	7
**ECMO / mechanical support**	4	8
**Low cardiac output (categorised)**	4	8
**Mental health consequences**	4	10
**Necrotising enterocolitis**	4	11
**Hospital acquired infection (graded/categorised)**	4	12
**Prolonged hospital length of stay**	4	13
**Acute kidney injury (graded)**	4	14
**Prolonged pleural effusion**	4	15
**Poor communication between clinical team and family**	4	16
**Recurrent laryngeal nerve palsy**	4	17
**Vascular thrombosis (graded)**	4	18
**Surgical bleeding (graded)**	4	19
**Complications during surgery**	4	20
**Complete heart block (categorised)**	4	21
**Questionable clinical team decision & diagnosis**	4	22
**Phrenic nerve injury**	4	23
**Level of support at home**	4	24
JET	25	25
Sensory neural deafness	26	26
Elevated pulmonary vascular resistance	27	27
Liver injury (graded)	27	27

For peer review only - http://bmjopen.bmj.com/site/about/guidelines.xhtml

Table 3 – The final list of 9 selected morbidities

Morbidities
Acute neurological event
Unplanned reoperation / re-intervention
Problems feeding (excluding NEC)
Need for renal replacement therapy (excluding ECMO)
Major adverse event
ECLS / ECMO
Necrotising enterocolitis (NEC)
Hospital acquired infection (graded/categorised)
Prolonged pleural effusion / chylothorax

For peer review only

For peer review only - http://bmjopen.bmj.com/site/about/guidelines.xhtml

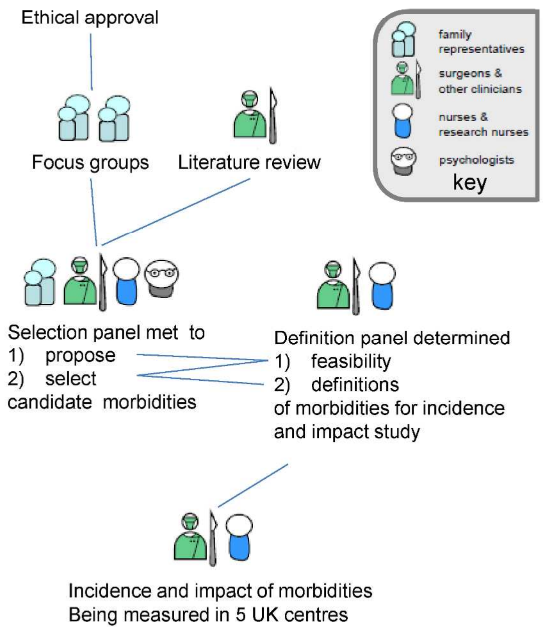

the role of the selection panel within our wider study to identify and measure the incidence and impact of important morbidities following paediatric cardiac surgery
Figure 1
254x190mm (300 x 300 DPI)

For peer review only

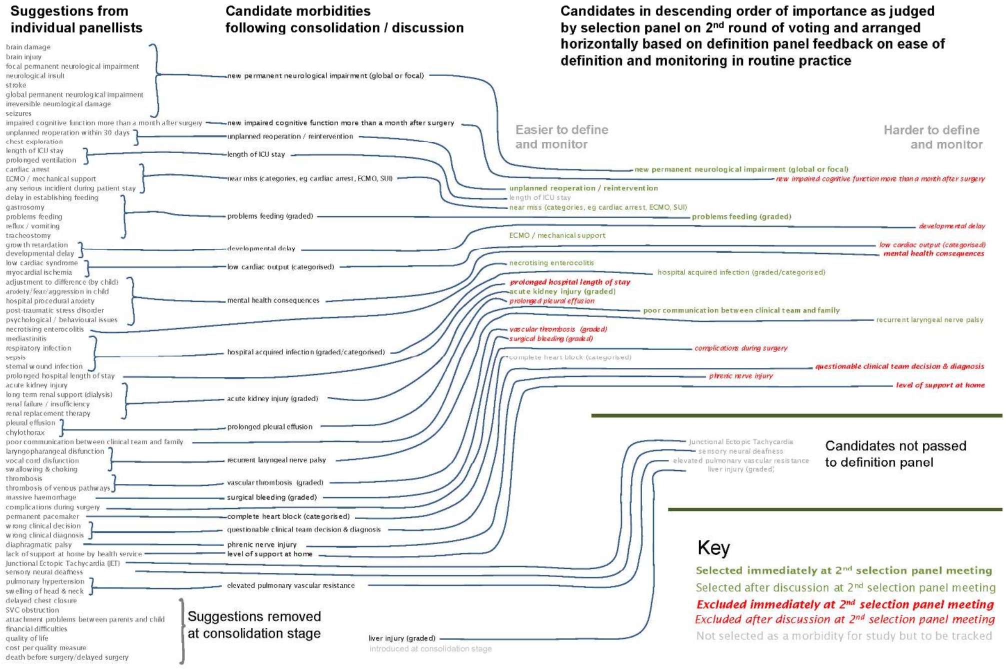

overview of results of selection process
Figure 2
254x190mm (300 x 300 DPI)

Appendix 1

Voting process used in selection

The voting process used as part of the nominal group technique deployed was based on the algorithm presented in *Utley M, Gallivan S, Mills M, Mason M, Hargraves C. A consensus process for identifying a prioritised list of study questions. Health Care Manag Sci. 2007 Feb;10(1):105–10*. A short summary is presented here.

In a secret ballot, each panellist ranked the candidate options in order of descending importance. They were permitted to use tied ranks and were not obliged to rank every option. For each and every possible pair of options (candidate morbidities A and B say) we determined whether at least as many participants preferred option A to option B as preferred option B to option A. We then used the analysis summarised in Box 1 to identify group preferences among the set of candidate morbidities.

In the first panel meeting, the rankings supplied by individual panellists were entered into an Excel spreadsheet and a VBA Macro written by MU and CP was used to conduct the analysis. For the online poll conducted after the second panel meeting, we used the web-based tool available at www.crankit.io.

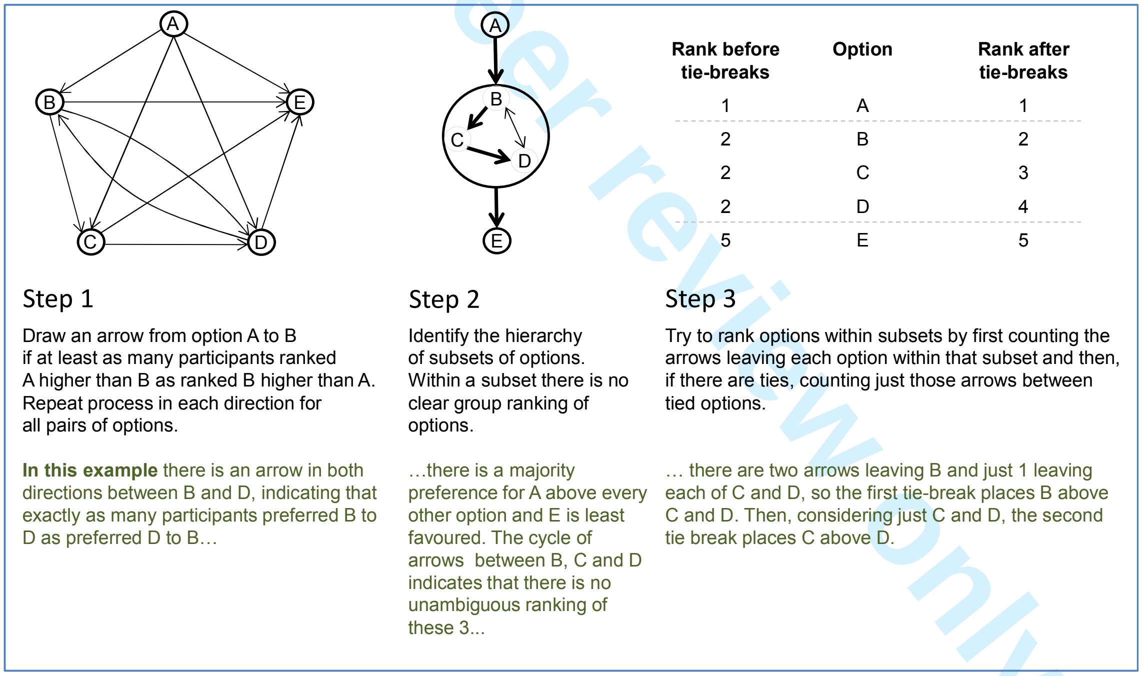

Box 1; explanation of the robust group ranking process used in the nominal group approach employed by the selection panel.

1

For peer review only

Appendix 2

Panel composition

Panellist	Role on panel
1*	Family representative
2	Heart surgeon
3†	Intensive care doctor
4†	Cardiologist
5	Intensive care doctor
6†	Paediatrician
7	Family representative
8*	Paediatrician
9	Intensive care nurse
10*	Heart surgeon
11	Family representative
12†	Clinical nurse specialist
13	Heart surgeon
14†	Cardiologist
15†	Clinical psychologist

Table A2 Members of the selection panel. * indicates members who could not attend the 1st panel meeting, † those who could not attend the 2nd meeting. Further details are available at http://www.gosh.nhs.uk/medical-information/clinical-specialties/cardiothoracic-surgery-information-parents-and-visitors/why-we-do-research/complications-after-heart-surgery-children.

For peer review only - http://bmjopen.bmj.com/site/about/guidelines.xhtml

Appendix 3: The initial 66 terms under consideration in selection panel 1.

anxiety/fear/aggression	prolonged ventilation
problems feeding	complications during surgery
reflux/vomiting	developmental delay
pleural effusion	financial difficulties
swallowing & choking	lack of support at home
global permanent neurological impairment	laryngopharyngeal dysfunction
Mediastinitis	vocal cord dysfunction
myocardial ischemia	delay in establishing feeding
focal permanent neurological impairment	brain damage
sensory neural deafness	low cardiac output
brain injury	Sepsis
cardiac arrest	gastrosomy
renal failure/insufficiency	massive haemorrhage
length of ICU stay	thrombosis
post-traumatic stress disorder	impaired cognitive function more than a month after surgery
ECMO / mechanical support	quality of life
communication	prolonged hospital length of stay
hospital procedural anxiety	permanent pacemaker
adjustment to difference	long term renal support
Attachment	any serious incident during patient stay
neurological insult	cost per quality measure
Chylothorax	seizures
acute kidney injury	stroke
necrotising enterocolitis	sternal wound infection
junctional ectopic tachycardia (JET)	psychological/behavioural issues
pulmonary hypertension	growth retardation
SVC obstruction	tracheostomy
swelling of head & neck	diaphragmatic palsy
chest exploration	delayed chest closure
thrombosis of venous pathways	respiratory infection
irreversible neurological damage	wrong clinical decision
renal replacement therapy	wrong clinical diagnosis
unplanned reoperation within 30 days	death before surgery/delayed surgery

For peer review only - http://bmjopen.bmj.com/site/about/guidelines.xhtml

Appendix 4 - The summary of the feasibility of defining and measuring in routine practice (as judged by the definition panel) vs importance shown to the selection panel at their second meeting.

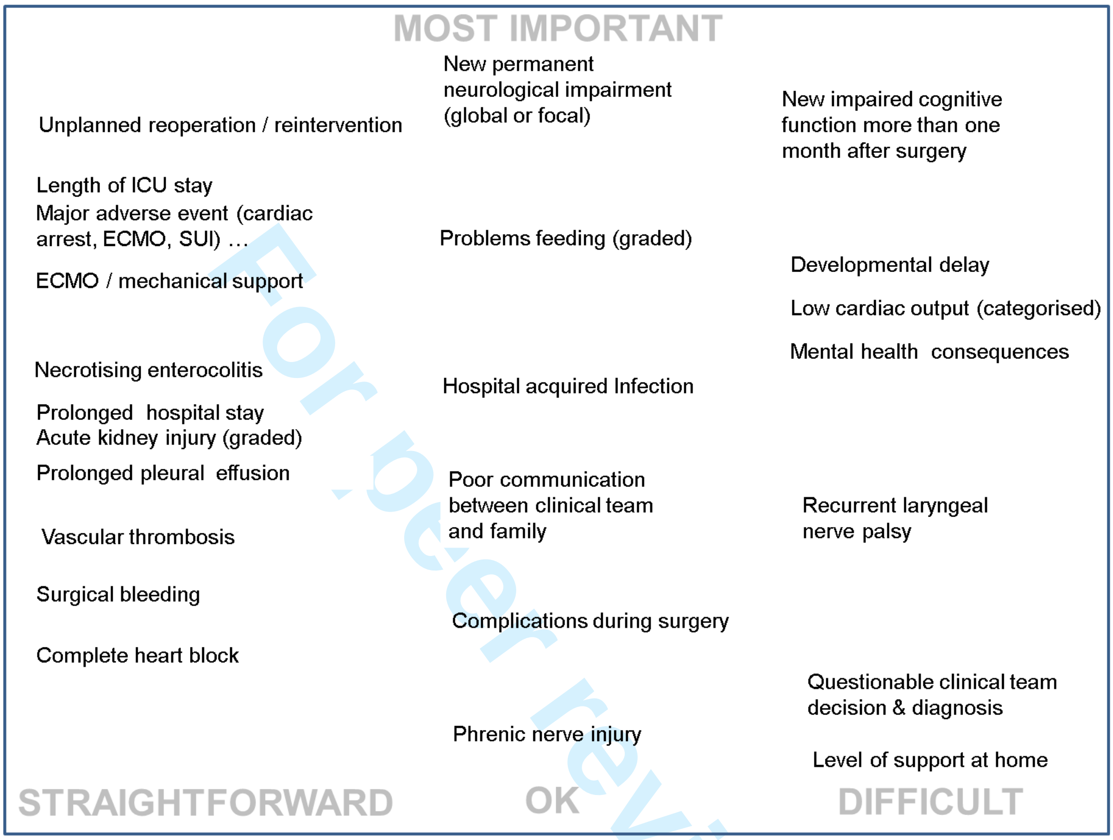

For peer review only

Appendix 5

Graphics shown to the selection panel at the second meeting on the relationships between candidate morbidities (figures A1-A3).

Figure A1

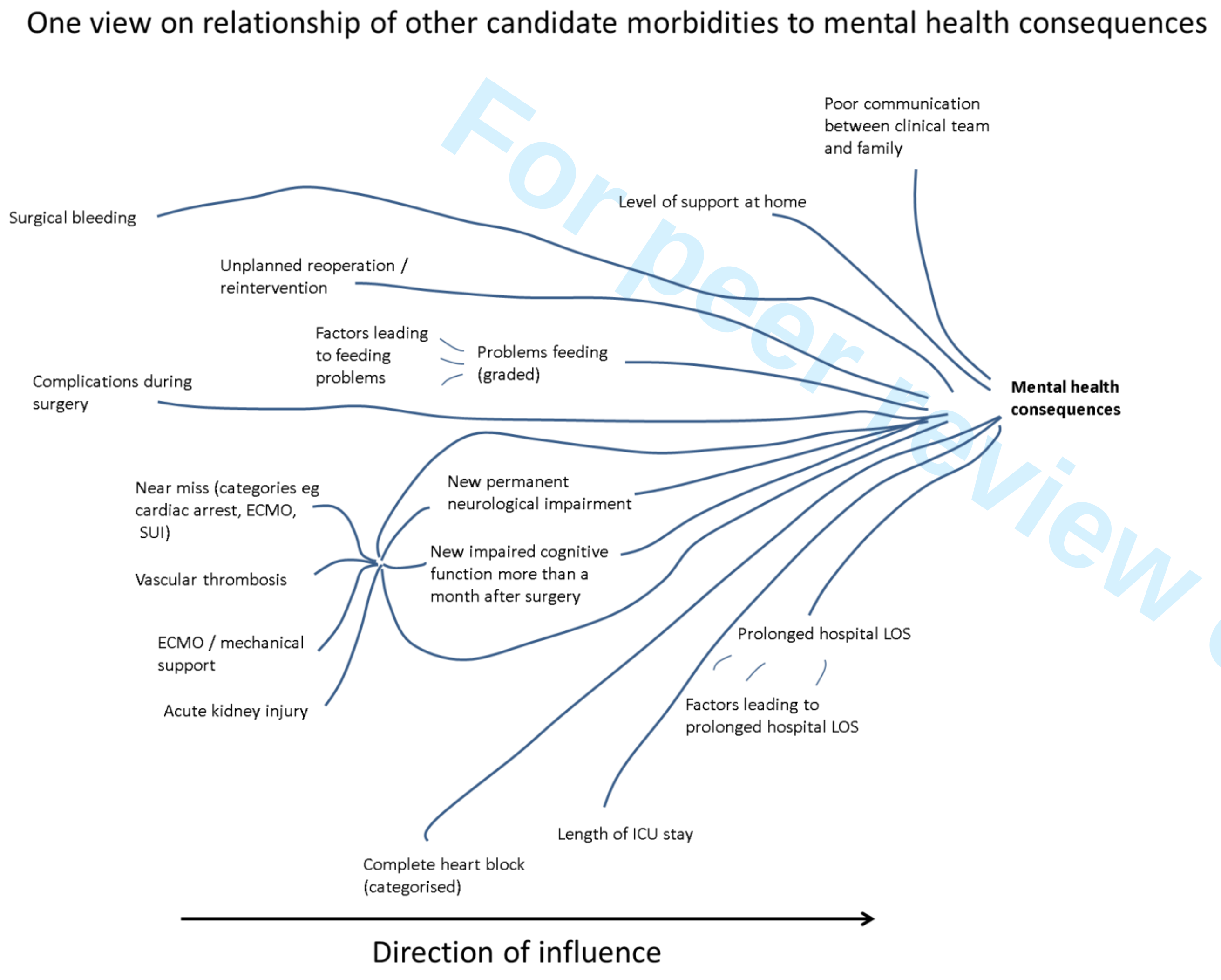

For peer review only - http://bmjopen.bmj.com/site/about/guidelines.xhtml

Figure A2

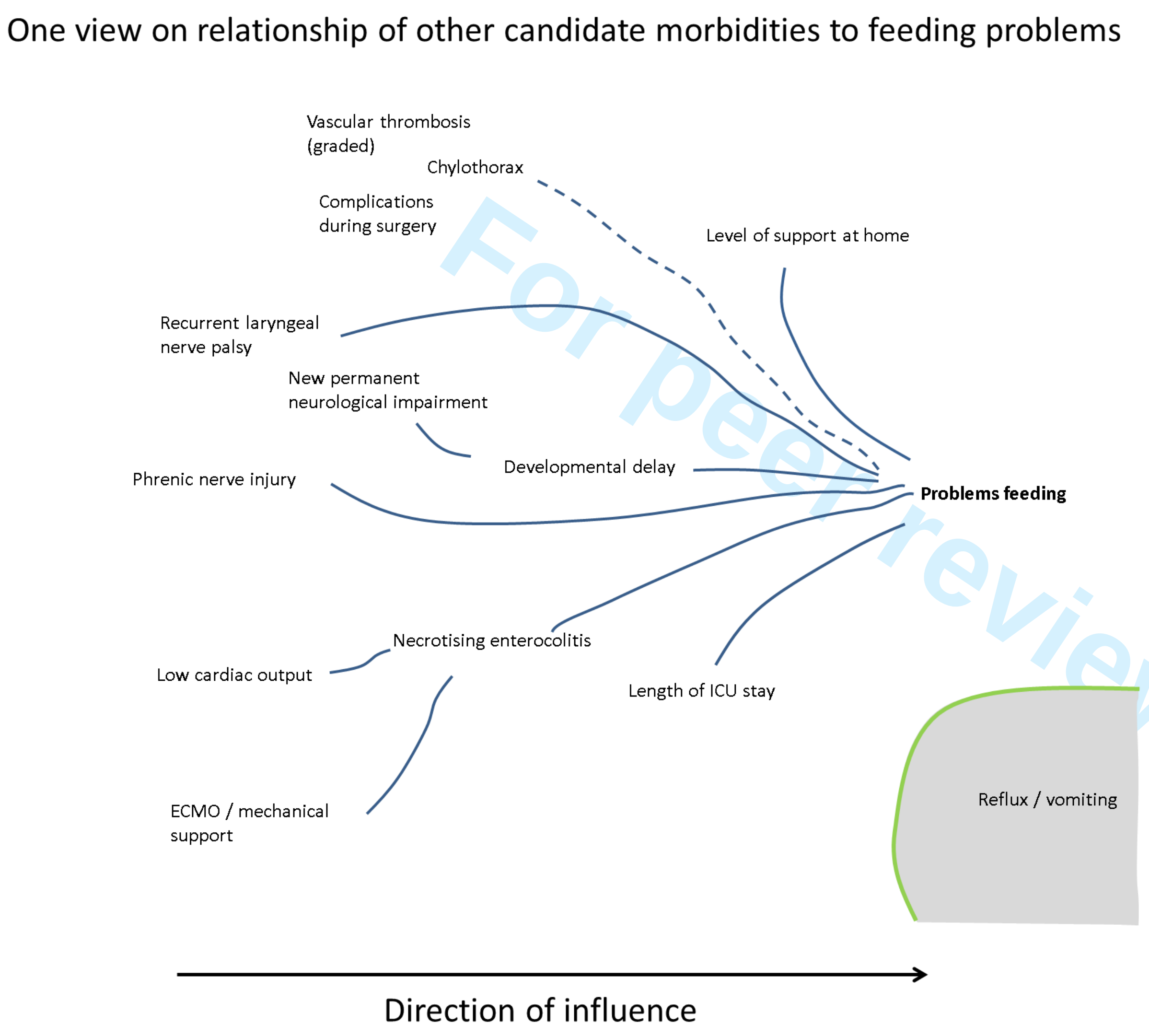

For peer review only

Figure A3

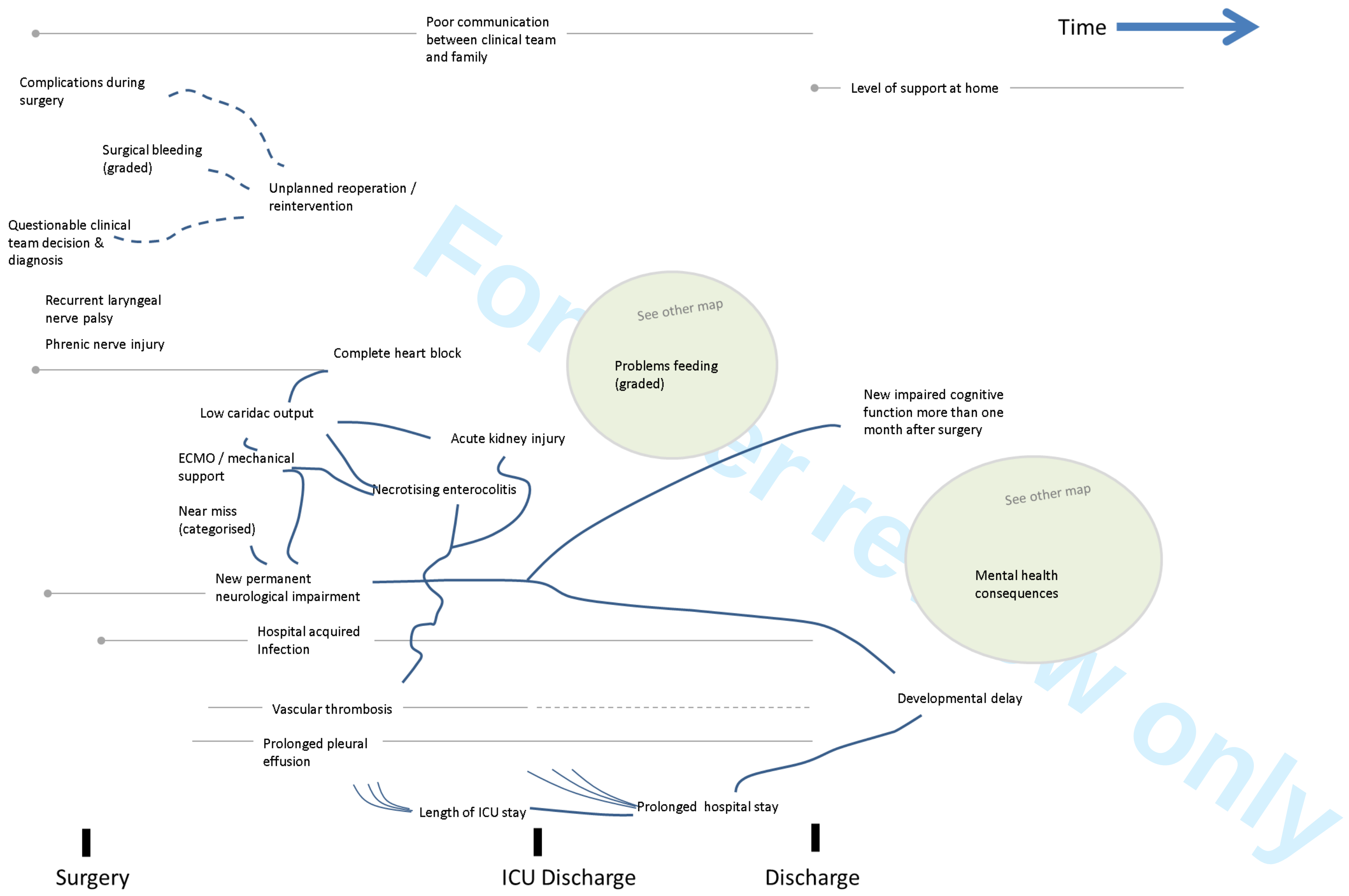

One view of whole picture (relationships of candidate morbidities to each other)

For peer review only
